# Macrophages in tumor cell migration and metastasis

**DOI:** 10.3389/fimmu.2024.1494462

**Published:** 2024-11-01

**Authors:** Madeline Friedman-DeLuca, George S. Karagiannis, John S. Condeelis, Maja H. Oktay, David Entenberg

**Affiliations:** ^1^ Integrated Imaging Program for Cancer Research, Albert Einstein College of Medicine/Montefiore Medical Center, Bronx, NY, United States; ^2^ Department of Pathology, Albert Einstein College of Medicine/Montefiore Medical Center, Bronx, NY, United States; ^3^ Montefiore Einstein Comprehensive Cancer Center, Albert Einstein College of Medicine/Montefiore Medical Center, Bronx, NY, United States; ^4^ Cancer Dormancy Institute, Albert Einstein College of Medicine/Montefiore Medical Center, Bronx, NY, United States; ^5^ Gruss-Lipper Biophotonics Center, Albert Einstein College of Medicine/Montefiore Medical Center, Bronx, NY, United States; ^6^ Department of Microbiology and Immunology, Albert Einstein College of Medicine/Montefiore Medical Center, Bronx, NY, United States; ^7^ Marilyn and Stanley M. Katz Institute for Immunotherapy of Cancer and Inflammatory Disorders, Albert Einstein College of Medicine/Montefiore Medical Center, Bronx, NY, United States; ^8^ Department of Surgery, Albert Einstein College of Medicine/Montefiore Medical Center, Bronx, NY, United States; ^9^ Department of Cell Biology, Albert Einstein College of Medicine/Montefiore Medical Center, Bronx, NY, United States

**Keywords:** cancer, macrophages, metastasis, tumor cell migration, chemotherapy, EMT

## Abstract

Tumor-associated macrophages (TAMs) are a phenotypically diverse, highly plastic population of cells in the tumor microenvironment (TME) that have long been known to promote cancer progression. In this review, we summarize TAM ontogeny and polarization, and then explore how TAMs enhance tumor cell migration through the TME, thus facilitating metastasis. We also discuss how chemotherapy and host factors including diet, obesity, and race, impact TAM phenotype and cancer progression. In brief, TAMs induce epithelial-mesenchymal transition (EMT) in tumor cells, giving them a migratory phenotype. They promote extracellular matrix (ECM) remodeling, allowing tumor cells to migrate more easily. TAMs also provide chemotactic signals that promote tumor cell directional migration towards blood vessels, and then participate in the signaling cascade at the blood vessel that allows tumor cells to intravasate and disseminate throughout the body. Furthermore, while chemotherapy can repolarize TAMs to induce an anti-tumor response, these cytotoxic drugs can also lead to macrophage-mediated tumor relapse and metastasis. Patient response to chemotherapy may be dependent on patient-specific factors such as diet, obesity, and race, as these factors have been shown to alter macrophage phenotype and affect cancer-related outcomes. More research on how chemotherapy and patient-specific factors impact TAMs and cancer progression is needed to refine treatment strategies for cancer patients.

## 1 Introduction

Metastasis – the systemic spread of cancer – causes the majority of cancer-related deaths (1). To metastasize, cancer cells must be able to migrate through the tumor microenvironment (TME) and intravasate. Though not all tumor cells are inherently capable of such feats, the migratory and invasive phenotypes needed to accomplish these tasks can be induced through interactions with other types of cells in the TME, including endothelial cells, immune cells, and fibroblasts. Of the cellular components within the TME, macrophages are key players in the induction of pro-metastatic phenotypes in cancer cells. In this review, we provide an introduction to macrophages and their origin, discuss macrophage polarization, and then review the latest understanding of the role of macrophages in tumor cell migration and metastasis, including the promotion of 1) epithelial-mesenchymal transition (EMT), 2) pro-tumoral extracellular matrix (ECM) remodeling, 3) tumor cell chemotaxis towards blood vessels, and 4) tumor cell intravasation. We also explore the impact of chemotherapy and host factors including diet, obesity, and race on tumor-associated macrophages (TAMs). Overall, we attempt here to summarize recent studies, discuss these new findings in the context of what is already known about the role of TAMs in tumor cell migration and metastasis, and highlight new potential avenues for refining therapeutic interventions.

### 1.1 Macrophage ontogeny

Macrophages have two distinct ontogenies. The first of these is monocyte-derived macrophages (MDMs) which originate from progenitors in the bone marrow and other hematopoietic niches (2), progress through several stages of differentiation, and enter the systemic circulation as monocytes. Circulating monocytes are recruited to tissues in response to locally released chemo-attractants where they differentiate into macrophages (3). Once inside the tissue, MDMs may be short- or long-lived, and their population is maintained through recruitment of new circulating monocytes as well as proliferation of pre-existing MDMs (4, 5). The second group, known as tissue-resident macrophages, arise early in embryonic development, migrating from the yolk sac or fetal liver into developing organs where they differentiate into tissue-specific macrophages, including Kupffer cells (liver), osteoclasts (bone), and microglia (brain) (5). In adults, these macrophages self-renew largely independently of the bone marrow (6, 7). Macrophages in the TME are referred to as tumor-associated macrophages (TAMs). While most TAMs are monocyte-derived, tissue-resident macrophages make up a considerable percentage of TAMs in some tumor types (8–10).

### 1.2 Macrophage polarization

Once inside the tumor, macrophages take on various phenotypes and functions in response to stimuli in the microenvironment. These phenotypes are referred to as “polarization states.” There is a wide spectrum of macrophage polarization states ranging from pro-inflammatory (M1) to anti-inflammatory (M2).

M1 macrophages (historically called “classically activated”), are pro-inflammatory cells that participate in the host immune response against pathogens and can have anti-tumor activity. As such, environmental factors associated with infection and inflammation (including interferon (IFN)-γ, bacterial lipopolysaccharide (LPS), and granulocyte-macrophage colony stimulating factor (GM-CSF)) promote M1 polarization (11). These signals cause macrophages to express surface proteins related to antigen presentation and T cell activation (including HLA-DR, CD80, and CD86) (11–14), and secrete inflammatory cytokines such as tumor necrosis factor (TNF)-α and interleukin (IL)-1β to further enhance the immune response (11). M1 macrophages promote tumor cell killing through strong antigen presentation and effective activation of the innate and adaptive immune responses (15). Indeed, high M1 macrophage infiltration is correlated with positive outcome in cancer patients (16, 17).

M2 (or “alternatively activated”) macrophages, are anti-inflammatory cells that are involved in tissue repair and immune suppression. While these cells are essential in maintaining homeostasis in healthy tissues, they can also promote tumor growth and metastasis in the TME. M2 macrophages are induced by anti-inflammatory cytokines in the microenvironment, including IL-4 and IL-10 (11). These signals cause macrophages to express surface proteins such as CD163 and CD206, which are involved in tissue “clean-up” and homeostasis, and to secrete additional anti-inflammatory factors, such as IL-10 and transforming growth factor (TGF)-β, which further suppress the immune response (11, 18, 19). M2 macrophages also express high levels of vascular endothelial growth factor (VEGF), which promotes tumor vascularization, enhancing the delivery of oxygen and nutrients to the tumor (11). M2 macrophages are poor antigen presenters and suppress both innate and adaptive anti-tumor immunity (15). Furthermore, M2 macrophages are implicated in chemoresistance and metastasis, and high M2 infiltration is associated with poor prognosis in cancer patients (20–26).

Though macrophage polarization is a spectrum with M1 and M2 on opposing ends, it is common in the literature to oversimplify this state and treat macrophage polarization as a dichotomy (M1 *or* M2). Given that many “anti-inflammatory” macrophages also participate in inflammatory signaling and vice versa, the terms “M1” and “M2” should merely give a sense of how a macrophage is predominantly functioning in a particular environment. It is also important to note that macrophage phenotype is highly plastic. Similar to other components of the innate immune system, macrophage phenotype can quickly change in response to environmental cues (27). Indeed, *in vitro* and *in vivo* studies confirm that macrophages may repolarize in response to particular stimuli (28–30), an effect that has been leveraged in several immunotherapy clinical trials (31). Promising macrophage-targeting therapies and the challenges associated with their development are reviewed elsewhere (32–37). Given their significant, plastic, and diverse roles in cancer progression, understanding the mechanisms behind macrophage-mediated cancer progression and the effects of chemotherapy and host factors is crucial to refining cancer treatment strategies.

## 2 TAMs in EMT

**Figure 1:**
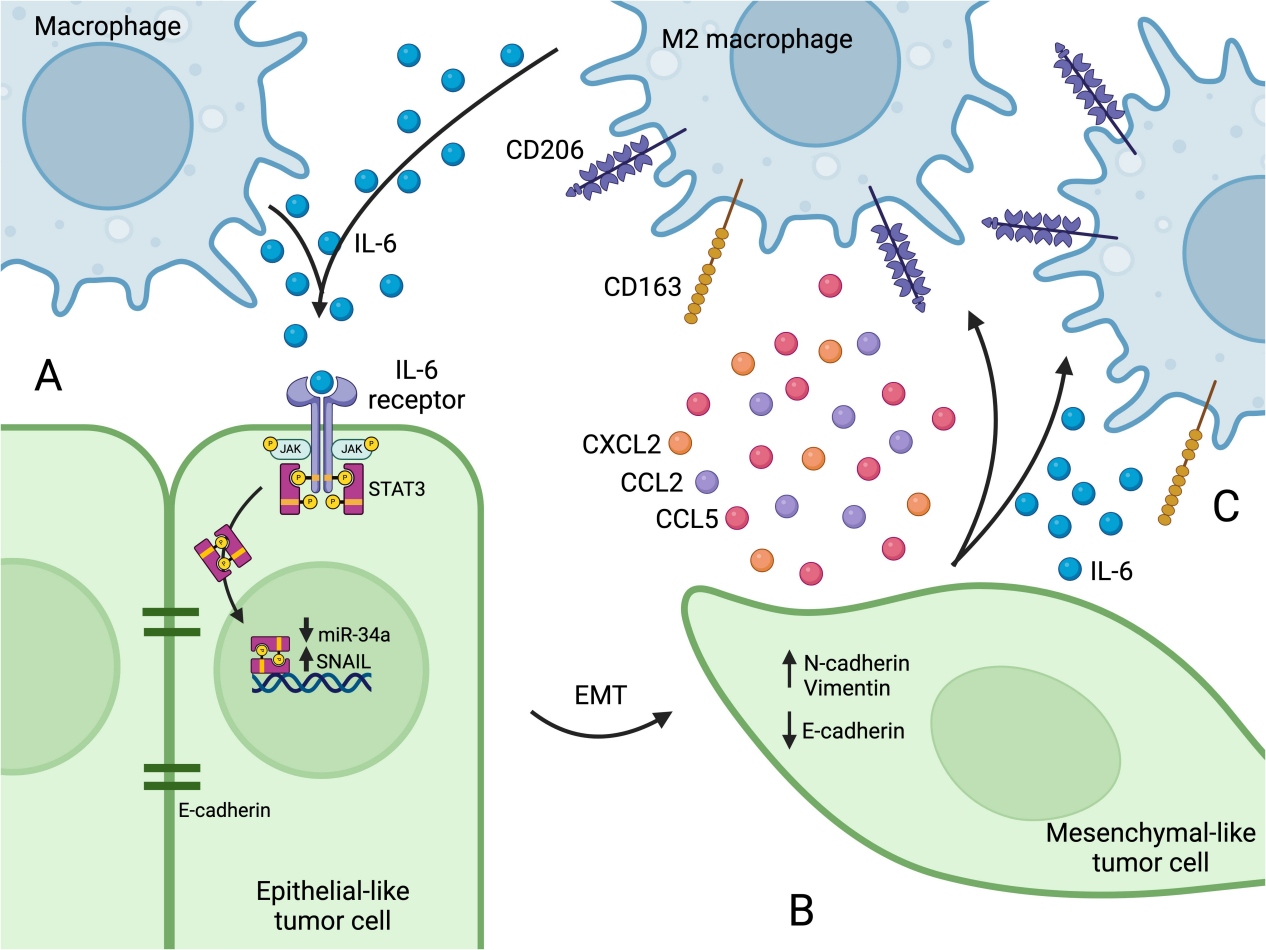
**(A)** Macrophages secrete IL-6, which binds to the IL-6 receptor on tumor cells, activating the JAK2/STAT3 pathway. After IL-6 receptor activation, STAT3 translocates to the nucleus and suppresses transcription of miR-34a, which leads to SNAIL upregulation. **(B)** The increase in SNAIL leads to loss of E-cadherin, and EMT programs become active and increase the expression of N-cadherin and vimentin. The tumor cell takes on a mesenchymal-like phenotype, which affords enhanced migration capacity. **(C)** Mesenchymal-like tumor cells secrete factors that recruit macrophages to the TME (e.g. CCL2, CCL5, and CXCL2) and that promote M2 polarization (e.g. IL-6). M2 polarization is characterized by the expression of surface markers such as CD163 and CD206. Figure created with BioRender.com.

Epithelial-mesenchymal transition (EMT) is the process by which epithelial cells lose their characteristic apical-basal polarity and tight cell-cell junctions, and gain features associated with mesenchymal cells, including the ability to migrate and invade surrounding tissue (38, 39). In healthy tissues, EMT is used in critical processes such as embryonic development and wound healing. However, cancer cells hijack this program to gain migratory and invasive phenotypes. Cells that have undergone EMT are characterized by the loss of E-cadherin and the increase in N-cadherin and vimentin. E-cadherin, often used as an epithelial cell marker, is an important cell-cell adhesion protein involved in contact-mediated inhibition of cell growth (40). During EMT, the transcription factor SNAIL directly represses E-cadherin transcription and is thus crucial in EMT regulation (41). As E-cadherin decreases, the mesenchymal cell markers N-cadherin and vimentin increase and support tumor cell survival and migration (42, 43). During this process, tumor cells pass through a series of epithelial/mesenchymal (E/M) hybrid states that reflect varying degrees of plasticity and metastatic potential (44). Tumor-associated macrophages have long been known to play a role in EMT induction (45, 46), and more recent evidence shows that TAMs also promote progression to later E/M hybrid states (44). A number of recent studies have further elucidated the mechanisms behind this relationship, pointing to feedback loops in which tumor cells undergoing EMT attract and polarize macrophages, which then secrete factors that further promote EMT in tumor cells (45–47) (**Figure 1**).

Macrophages can induce EMT in cancer cells by secreting various factors, including TGF-β (48), CCL2 (49), and IL-6 (50), all of which ultimately lead to SNAIL upregulation and subsequent EMT in tumor cells. For instance, IL-6 activates the JAK2/STAT3 pathway upon binding the IL-6 receptor (47, 50, 51) (**Figure 1A**). The JAK2/STAT3 axis is a critical signal transduction pathway that participates in many cellular functions including proliferation, differentiation, and survival, and components of this pathway are hyperactivated in many cancers (52, 53). After IL-6 receptor activation, STAT3 inhibits the transcription of tumor suppressor microRNAs including miR-34a (50, 51). MiR-34a suppresses SNAIL, and loss of miR-34a leads to SNAIL upregulation and subsequent EMT (54, 55), as well as tumor cell proliferation and migration (50, 56) (**Figure 1B**). Macrophage-induced mesenchymal-like tumor cells then secrete increased amounts of CCL2, which recruits macrophages (47), and IL-6, which leads to M2 polarization (51, 57) (**Figure 1C**), further propagating EMT in a positive feedback loop.

These studies reveal several therapeutic targets with the potential to reduce the co-induction of EMT and M2 polarization. Inhibiting IL-6 signaling by targeting IL-6 itself, or its receptor (with anti-IL-6R monoclonal antibodies like tocilizumab), reduces EMT, decreases M2 polarization and increases M1 polarization (51). The tumor suppressor miR-34a is also a potential target. MiR-34a suppresses SNAIL and reinstates an epithelial phenotype in mesenchymal-like cancer cells (55). MiR-34a expression in tumor cells also promotes macrophage M1 polarization, demonstrating that the microRNA can favorably modify both the tumor cells and the immune microenvironment (51). Indeed, nanoparticle-delivered miR-34a has shown promise in treating several types of cancers (58, 59).In addition to promoting EMT in cancer cells, SNAIL is also involved in macrophage recruitment. SNAIL expression in tumor cells increases their secretion of CCL2, CCL5, and CXCL2, all of which attract macrophages to the TME (60–62) (**Figure 1C**). Indeed, SNAIL-overexpressing tumors show a significant increase in macrophage infiltration, M2 polarization, and metastasis (60, 62).

Finally, in addition to cytokines and chemokines, more recent evidence has revealed that exosomes can also mediate macrophage-tumor cell feedback loops related to EMT and M2 polarization. Tumor cells that have undergone EMT secrete exosomes containing microRNAs that are taken up by macrophages and induce M2 polarization (63). For example, it was shown that tumor cell derived exosomes contain miR-106b-5p, which upon uptake by macrophages, activates the PI3Kγ/AKT/mTOR signaling pathway to induce M2 polarization by downregulating the pathway inhibitor PDCD4 (63). Similarly, SNAIL expression directly upregulates the transcription of miR-21 in tumor cells. This microRNA is then transferred to macrophages through exosomes and also targets macrophage PDCD4, leading to M2 polarization (64).

Exosomal microRNAs can be transmitted from macrophages to tumor cells as well (65). Tumor cell uptake of M2 macrophage-derived exosomes leads to downregulation of E-cadherin and upregulation of N-cadherin and vimentin (65). Lu et al. found that these exosomes contain miR-23a-3p, which downregulates the tumor suppressor PTEN (65) – a known regulator of EMT (66). In a positive feedback loop, tumor cells treated with M2 macrophage-derived exosomes express higher levels of CCL2, leading to increased macrophage recruitment and M2 polarization (65).

In summary, tumor cell EMT and macrophage recruitment and polarization are intimately connected and co-regulated by several molecular mechanisms.

## 3 TAMs in extracellular matrix remodeling

### 3.1 TAMs in matrix stiffness

Macrophages also promote tumor cell migration and invasion through ECM remodeling. The ECM of non-cancerous soft tissue is characterized by “curly,” non-dense collagen fibers that lay parallel to the epithelium (67). This soft ECM is involved in maintaining an epithelial phenotype, and matrix stiffening has been shown to play a direct role in promoting EMT (68, 69). Indeed, clinical conditions characterized by a stiff ECM – including cirrhosis of the liver (70), pulmonary fibrosis (71), and mammographically dense breast tissue (72) – are associated with a higher incidence of cancer in the respective tissues. In tumors, collagen deposition increases, and fibers become stiff, cross-linked and linearized – a process known as desmoplasia that has been associated with immune evasion and metastasis (67, 73–76). Indeed, tumors have been shown to be stiffer than healthy tissue in breast (77), pancreas (78), bladder (79), and ovarian (80) cancers. The stiffened matrix of tumors promotes malignant transformation, proliferation, and invasion of tumor cells, and acts as a “highway,” guiding tumor cells towards the vasculature, where they further invade and intravasate (67, 69, 76, 77, 81–85). Recent work sheds light on the mechanistic role of TAMs in pro-tumoral matrix stiffening.

Macrophages promote matrix deposition and stiffening in both cancerous and healthy tissue (86, 87). In pancreatic cancer, macrophages foster desmoplasia indirectly by activating pancreatic stellate cells. Mechanistically, macrophages internalize and degrade surrounding collagen, which leads to an increase in inducible nitric oxide synthase (iNOS) and the production of reactive nitrogen species (RNS). RNS then activate pancreatic stellate cells leading to increased collagen deposition and desmoplasia (87).

In the desmoplastic reaction, excessive ECM deposition is followed by cross-linking, which confers increased stiffness to the TME. ECM crosslinking is mediated primarily by lysyl oxidase (LOX) and lysyl oxidase-like (LOXL) proteins, which are expressed by a variety of cells in the TME (88). In pancreatic cancer, LOXL2 expression is positively associated with tumor burden and metastasis (89). Macrophages both express their own LOXL2 and promote its expression in tumor cells (89, 90). Alonso-Nocelo et al. recently demonstrated that macrophage depletion leads to a significant decrease in LOXL2, collagen fibril orientation, and metastasis in mice, indicating that macrophages promote matrix stiffness (89). In a positive feedback loop, the stiffened matrix then promotes macrophage infiltration and M2 polarization (89). Mechanistically, macrophages increase matrix stiffness by secreting oncostatin M (OSM), which upregulates LOXL2 in tumor cells (89). In turn, the stiffened matrix activates integrin β5 in macrophages, leading to FAK/MEK/ERK activation and LOXL2 upregulation, further supporting ECM crosslinking in the TME (90). In addition to promoting tumor cell migration, stiffened matrices cause macrophages to take on a more immunosuppressive phenotype (89, 91). Indeed, macrophages cultured on stiff matrices recruit cytotoxic T cells less efficiently than those cultured on softer matrices (91).

Together, these studies indicate that macrophages support the development of a stiff ECM through direct and indirect mechanisms. In turn, the stiff ECM promotes macrophage recruitment, M2 polarization, tumor cell migration, and metastasis.

### 3.2 TAMs in matrix degradation

Equally as important as matrix stiffness for cancer progression is matrix degradation, which is mediated by matrix metalloproteinases (MMPs). MMPs are a group of zinc-containing proteolytic enzymes responsible for degrading the extracellular matrix (92). MMPs are upregulated in nearly every type of cancer, and their activity has been shown to facilitate angiogenesis, tumor cell immune evasion, migration, and metastasis (92, 93). MMPs are expressed by a variety of stromal, immune, and tumor cells, and a growing body of evidence reveals the role of MMPs in the dynamic pro-metastatic interplay between macrophages and tumor cells.

MMP production can be induced through several major signal transduction pathways including STAT3, ERK, and NF-κB (94–102). Evidence shows that macrophages provide multiple ligands for these pathways that cooperate to promote MMP expression. For example, macrophages secrete AEG-1, TGF-β, and IL-6, which all increase MMP-9 expression in tumor cells by activating STAT3 (94, 95, 97). Indeed, inhibiting STAT3 in tumor cells, or its activators in macrophages, causes a significant decrease in MMP expression and migration in tumor cells (95, 97). Macrophages also secrete TNF-α and IL-1β, which activate the NF-κB pathway. Yamanaka et al. found that IL-1β activates NF-κB in gastric cancer cells, and this leads to increased MMP-9 expression and tumor cell invasion (103). Furthermore, tumor cells cultured in M2 macrophage-conditioned media express significantly increased levels of MMP-9 (98). This effect can be seen to a lesser (but still significant) extent when tumor cells are stimulated with TNF-α alone, suggesting that macrophages provide multiple ligands that stimulate MMP production (98).

MMPs expressed by macrophages also play a significant role in tumor cell invasion and metastasis. Macrophage – but not tumor cell – expression of MMP-11 is a negative prognostic marker in breast cancer (104). MMP-11-overexpressing macrophages secrete increased amounts of CCL2. CCL2 then activates MAPK signaling in tumor cells and increases tumor cell migration and MMP-9 expression (104). In Wilms’ tumor and gastric cancer, MMP-9 is upregulated in M2 macrophages, and MMP-9 initiates EMT and increases tumor cell invasion (105, 106). Mechanistically, macrophage-derived MMP-9 activates the PI3K/AKT pathway in tumor cells leading to the upregulation of SNAIL and subsequent EMT (105, 106). These studies identify MMP-9 as a promising therapeutic target. Indeed, MMP-9 inhibition increased the efficacy of chemotherapy and decreased metastasis to the lungs in a mouse model of gastric cancer (106). Together, these studies identify macrophages as important regulators of tumor cell MMP production.

## 4 TAMs in tumor cell chemotaxis

**Figure 2:**
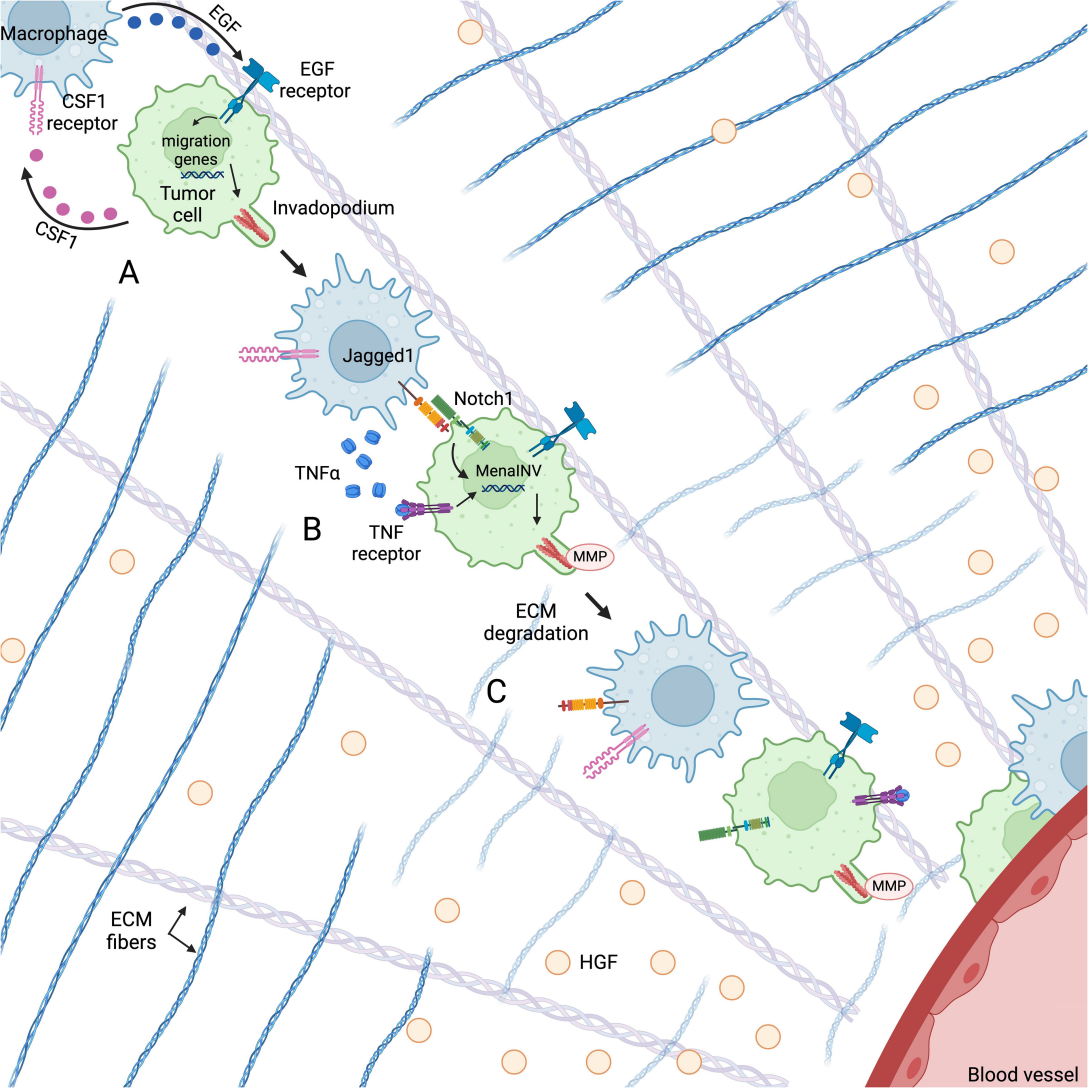
Macrophages and tumor cells co-migrate through the TME along fibronectin-collagen I ECM fibers towards HGF gradients secreted by endothelial cells using an EGF/CSF1 paracrine loop. **(A)** Macrophage-derived EGF activates the EGF receptor on the tumor cell, leading to the upregulation of genes associated with cell migration and invadopodium formation. **(B)** Cooperative Notch1/NF-κB signaling between the macrophage and tumor cell leads to an increase in MenaINV expression, which enhances invadopodium stability and degradative activity. **(C)** The invadopodium degrades ECM in its path, facilitating tumor cell migration towards the blood vessel. Figure created with BioRender.com.

Beyond promoting a mesenchymal phenotype in cancer cells, TAMs also supply ligands and chemotactic factors that support tumor cell migration and invasion in the tumor microenvironment (**Figure 2**).

*In vitro* and *in vivo* migration assays and intravital imaging show that tumor cells and macrophages migrate through the TME together using a CSF1/EGF paracrine loop that leads to invasion and metastasis (107–110) (**Figure 2A**). High levels of CSF1 (111–113) and the EGF receptor (114–117) are correlated with metastasis and poor prognosis in a number of solid tumors. CSF1 secreted by tumor cells both recruits macrophages to the tumor microenvironment and promotes macrophage expression of EGF (108). TAM-derived EGF then binds to the EGF receptor on tumor cells, leading to increased CSF1 production and activation of pathways associated with migration (107, 108, 118) (**Figure 2A**). Using this paracrine loop, macrophages and tumor cells migrate together along fibronectin-collagen I ECM fibers towards chemotactic gradients. Leung et al. found that in the TME, the primary chemo-attractant for the macrophage-tumor cell pair is hepatocyte growth factor (HGF), which is secreted by endothelial cells (119) (**Figure 2**). Within 500 μm of a blood vessel, tumor cells may perform sustained directional migration towards HGF gradients with or without macrophages. However, tumor cells at greater distances can only move towards blood vessels by co-migrating with a macrophage (119). Thus, while tumor cells may chemotax along these HGF gradients alone, co-migrating with macrophages greatly supports their ability for sustained directional migration and extends the chemoattractive influence of the blood vessels.

In addition to participating in tumor cell chemotaxis, macrophages support tumor cell migration by both promoting the formation of tumor cell invadopodia and prolonging their activity (**Figure 2**). Invadopodia are F-actin-rich protrusions with MMP activity used to degrade the ECM and create new physical pathways through the tumor (120, 121). TAMs promote the formation of invadopodia by secreting EGF, which activates the EGF receptor in tumor cells. EGF receptor activation initiates the assembly of invadopodial precursors through the recruitment of actin regulatory proteins such as cortactin, Arp2/3, and cofilin (118, 120, 122, 123) (**Figure 2A**). Phosphorylation of cortactin activates actin polymerization and leads to maturation of a precursor. The actin regulatory protein Mena (encoded by the *ENAH* gene) supports this polymerization by localizing to the barbed ends of polymerizing actin filaments and temporarily interfering with the capping proteins that block polymerization (124, 125). In non-invasive tumor cells, invadopodia can form, but do not mature, as cortactin is rapidly dephosphorylated by the tyrosine phosphatase PTP1B that is constitutively bound to Mena. This lack of maturation dramatically limits the invasive capacity of invadopodia by limiting the amount of matrix they can degrade (126).

Macrophages also play a role in promoting invadopodium maturation (thus increasing degradative activity) by stimulating the expression of a splice variant of Mena called MenaINV (**Figure 2B**). MenaINV prolongs the degradative activity of invadopodia by sequestering PTP1B and preventing the dephosphorylation/deactivation of cortactin and the subsequent disassembly of actin filaments (127, 128). The MenaINV-stabilized invadopodium then degrades the ECM in its path, facilitating tumor cell migration towards blood vessels (**Figure 2C**). Macrophages promote the alternative splicing of Mena through cooperative Notch1/NF-κB signaling (129, 130) (**Figure 2B**). Mechanistically, macrophages secrete TNFα, which activates the NF-κB pathway in tumor cells, leading to p65 nuclear translocation. Inside the nucleus, p65 binds to the κB binding sites on the *ENAH* promoter, initiating *ENAH* transcription. Macrophages also express the Notch1 ligand Jagged1, which engages the Notch1 receptor on tumor cells, causing nuclear translocation of the Notch1 intracellular domain (NICD). Nuclear NICD enhances the nuclear retention of p65, leading to sustained *ENAH* transcription and to alternative splicing (130). Prior work has shown that this alternative splicing is the switch that turns non-invasive tumor cells into invasive tumor cells (131).

In summary, macrophages partner with tumor cells to enhance directional migration and metastasis by guiding tumor cells towards blood vessels and promoting the assembly and invasion capacity of tumor cell invadopodia.

## 5 TAMs in tumor cell intravasation

Intravasation – the process by which tumor cells enter the vasculature – represents a key step in the metastatic cascade. Macrophages not only assist with tumor cell intravasation but are crucial for the process.

Breast cancer cells disseminate from the primary tumor through tumor microenvironment of metastasis (TMEM) doorways (132, 133). TMEM doorways are stable, tri-cellular structures (occurring primarily at vascular branch points) composed of a Mena-expressing tumor cell, a perivascular Tie2^High^ macrophage, and an endothelial cell in direct physical contact (132, 134–137). TMEM doorway density (hereafter referred to as TMEM doorway score) in the primary breast tumor is a clinically validated prognostic marker of distant metastasis (136, 138). Arwert et al. investigated the process of TMEM doorway assembly by systemically depleting macrophages and then tracking the fate of newly-recruited monocytes in the TME (139). They found that upon extravasation into the TME, monocytes become motile TAMs that begin to express CXCR4 and are then recruited back to the perivascular space by CXCL12-expressing perivascular fibroblasts. Once at the blood vessel, these motile TAMs become sessile, forming TMEM doorways with adjacent tumor and endothelial cells (139). Signaling between the three TMEM doorway cells results in the release of vascular endothelial growth factor-A (VEGFA) (132). The secreted VEGFA leads to the dissociation of local vascular endothelial cell-cell junctions, which causes a transient, localized vascular permeability event. Harney et al. used real-time multiphoton intravital imaging of a murine mouse model of breast cancer to show that these transient vascular permeability events are regulated and occur concurrent with tumor cell intravasation (132). Neither transient vascular permeability nor tumor cell intravasation occurs away from TMEM doorways (132). Furthermore, TMEM doorway score increases concomitantly with circulating tumor cells, and macrophage depletion leads to a significant reduction in TMEM doorways, vascular permeability, and circulating tumor cells, highlighting the essential role of TMEM doorway-associated macrophages in tumor cell intravasation (132, 133).

*In vitro* and *in vivo* studies confirm that invadopodia formation is necessary for tumor cell intravasation (140–143). In addition to initiating invadopodium formation through paracrine EGF signaling, macrophages can also initiate this process through direct contact (129, 140). In TMEM doorways, contact between the TMEM doorway-associated macrophage and tumor cell induces invadopodium formation in the tumor cell (129, 140). The invadopodium then degrades the basement membrane surrounding the vascular endothelium and functionally “holds the door open” for other migratory tumor cells to enter the blood stream (129, 140). Mechanistically, macrophage-tumor cell contact activates RhoA signaling in tumor cells, which initiates invadopodium formation in the tumor cell (140, 144). Indeed, RhoA knockdown reduces tumor cell invadopodium formation, matrix degradation, and intravasation (140, 144).

Importantly, targeting TMEM doorway-associated macrophages with the Tie2 inhibitor rebastinib has shown therapeutic promise by decreasing TMEM doorway function and metastasis in preclinical studies of breast cancer and pancreatic neuroendocrine tumors (133, 145). Mice treated with rebastinib have significantly reduced TMEM doorway activity, circulating tumor cells, and metastases compared to mice treated with vehicle control (133, 145).

Together, these studies identify macrophages as key mediators of tumor cell intravasation and demonstrate that blocking crucial macrophage signaling pathways may be a strategy to block tumor cell dissemination in patients.

## 6 TAMs in response to chemotherapy

Cytotoxic chemotherapies are characterized by their ability to directly prevent proliferation and promote apoptosis of dividing cells. Chemotherapeutic agents, including anthracyclines, platinum-based drugs, and other alkylating agents induce apoptosis by damaging DNA and preventing DNA replication and repair (146). Taxanes and vinca alkaloids prevent cell division by interfering with the mitotic spindle, and antimetabolites – structural analogs of nitrogenous bases – prevent DNA synthesis by getting fraudulently inserted into replicating DNA, as well as by preventing the synthesis of proper bases (146). Increasing evidence suggests that many of these drugs also exert indirect effects by modulating the immune microenvironment. While many of these indirect effects support tumor cell killing, some promote drug resistance and metastasis. In this section, we review the current understanding of how common chemotherapies affect macrophages in anti- and pro-tumoral ways.

### 6.1 Anti-tumor TAM response to chemotherapy

Paclitaxel – a microtubule stabilizing agent in the taxane group – has been shown to increase the immune response and tumoricidal activity of murine macrophages. In mice, paclitaxel treatment leads to a robust increase in macrophage expression of TNFα and IL-1β, pro-inflammatory cytokines associated with the M1 phenotype (147–149). Paclitaxel also increases macrophage expression of IL-12, a Th1-type cytokine involved in activating the innate and adaptive immune response (148, 150). As a LPS mimetic, paclitaxel activates toll-like receptor (TLR) 4 on murine macrophages leading to NF-κB activation and increased production of pro-inflammatory signals (148). Though some studies show that paclitaxel also activates TLR4 in human cells (151–153), others show that species-specific differences in the TLR4 accessory protein, myeloid differentiation factor 2 (MD-2), do not allow this activation (154–159). Interestingly, some studies indicate that docetaxel – another taxane – has more potent effects on human macrophages than paclitaxel. Millrud et al. found that docetaxel, but not paclitaxel, promoted an M1 phenotype in human monocytes (160). Furthermore, a clinical study assessing immune responses to taxanes in breast cancer patients showed that, while both docetaxel and paclitaxel lead to an increase in serum M1-associated markers (including IL-6, GM-CSF, and IFN-γ), the effects were significantly more pronounced in patients who received docetaxel (161). The effects of taxanes on macrophages are also highly context dependent. For instance, IFN-γ has been shown to “prime” macrophages for tumoricidal activity, and paclitaxel affords macrophages increased cytotoxicity after macrophage exposure to IFN-γ (162). While the exact mechanisms have yet to be elucidated, these studies indicate that taxanes can promote anti-tumor M1 polarization in a context-dependent manner.

In addition to taxanes, platinum-based drugs, antimetabolites, and alkylating agents have also been shown to promote M1 macrophage polarization. The combination of platinum-based agents with antimetabolites is a common first-line treatment for gastric cancer (163). In studies analyzing the TME of matched pre- and post-treatment biopsies from gastric cancer patients, post-treatment samples harbored significantly more M1-polarized macrophages, and this increase was associated with a favorable response to treatment (164, 165). Furthermore, when given at high doses, the alkylating agent cyclophosphamide is highly immunosuppressive. However, lower doses of the drug strikingly improve anti-tumor immunity (166). This has led to the development and use of metronomic schedules of administration, in which low doses of the drug are administered more frequently (166). In line with observations that low-dose cyclophosphamide improves anti-tumor immunity, several studies show that low-dose, metronomic cyclophosphamide increases macrophage M1 polarization and decreases tumor burden (167–169).

Leukemia and lymphoma are often treated with monoclonal antibodies. While these treatments are largely effective at targeting cancer cells in many niches, cancer cells often become resistant to such antibodies in the bone marrow (170–172). Several studies found that combining antibody therapy with cyclophosphamide prevented antibody therapy resistance in the bone marrow in part by promoting macrophage phagocytosis of antibody-targeted cancer cells (170–172).

In summary, these studies show that in some circumstances, chemotherapy reprograms macrophages to increase anti-tumor activity.

### 6.2 Pro-tumor TAM response to chemotherapy

The macrophage response to chemotherapy is a double-edged sword. While some studies show that chemotherapy promotes the anti-tumor activity of macrophages, others show that chemotherapy causes a macrophage-mediated pro-tumoral response.

Chemotherapy causes tumor cell death and tissue damage followed by a cytokine storm that promotes the release of endothelial and immune progenitor cells from the bone marrow (173–175). In response to this tissue damage, cells in the TME initiate a wound healing response by increasing their secretion of CSF1, CXCL12, and other chemokines that recruit these circulating progenitor cells to the tumor (176, 177). One result of this response is that perivascular TAMs increase following chemotherapy (133, 177). These newly recruited perivascular TAMs express high levels of VEGFA and the angiopoietin receptor Tie2, which have been shown to promote relapse and metastasis following chemotherapy (132, 133, 177–180). Several studies show how chemotherapy induces a macrophage-mediated pro-tumoral effect that can be abrogated by targeting macrophages.

Hughes et al. used mouse models of breast cancer to show that treatment with cyclophosphamide causes an increase in CXCR4-expressing perivascular macrophages, which promote tumor revascularization and regrowth via VEGFA signaling (177). Blocking CXCR4 signaling prevents the accumulation of perivascular macrophages and delays tumor regrowth (177).

In neuroblastoma, chemotherapy leads to the selective expansion of CCL2-expressing mesenchymal-like tumor cells and macrophage infiltration in patients, which promotes relapse and chemo-resistance (181). In mouse models, combining chemotherapy with CSF1R inhibition prevents macrophage infiltration and tumor regrowth (181).

Furthermore, while chemotherapy increases the infiltration of Tie2+ macrophages, Tie2 inhibitors have been shown to work synergistically with chemotherapy to delay tumor growth (145) and relapse (182).

In addition to promoting tumor relapse, macrophages can also increase tumor cell dissemination following chemotherapy. We have previously shown that treatment with paclitaxel causes a robust, macrophage-dependent increase in MenaINV expression, which promotes tumor cell migration, intravasation, and metastasis (130, 133). This indicates that paclitaxel causes a macrophage-mediated increase in metastasis-competent tumor cells, though the exact mechanism behind this effect remains unknown.

Furthermore, chemotherapy significantly increases the assembly and function of TMEM doorways, which are portals for tumor cell intravasation. Indeed, circulating tumor cells and lung metastases are more prevalent in mice treated with paclitaxel compared to vehicle control (133). Concerningly, TMEM doorway assembly is also increased in patients with ER+/HER2- breast cancer following neoadjuvant chemotherapy (133), thus increasing their risk of distant metastasis (136). As mentioned in Section 5, targeting TMEM doorway-associated macrophages with the Tie2 inhibitor rebastinib dramatically decreases the pro-metastatic effects of chemotherapy in pre-clinical studies, indicating that Tie2 inhibition in combination with a cytotoxic agent may improve patient outcomes (133, 145).

Another mechanism by which TAMs promote metastasis in response to chemotherapy is by upregulating the enzyme heparanase. Heparanase cleaves heparan sulfate, which is an important structural component of the ECM (183). Similarly to MMPs, this matrix-degrading enzyme is upregulated in many cancers and correlates with increased metastasis and poor prognosis (184). Unfortunately, heparanase has been shown to increase in some patients following chemotherapy (185). Mechanistically, treatment with chemotherapy leads to an increase in VEGFR3-expressing TAMs which secrete cathepsins that activate heparanase and promote ECM remodeling, lymphangiogenesis, and metastasis (186). Notably, blocking VEGFC/VEGFR3 signaling inhibits chemotherapy-induced lymphangiogenesis and metastasis (186).

In summary, chemotherapy can act on macrophages to promote relapse and metastasis in a variety of ways. Recent pre-clinical studies show that targeting macrophage recruitment or function is a promising approach to optimize cancer treatment. Indeed, there has been a 3-fold increase in clinical trials on macrophage-targeted therapies in the past 10 years (31). However, due to the diversity of patients, chemotherapies, and macrophage phenotypes, more research is needed to clarify the exact mechanisms of chemotherapy-induced cancer progression to refine treatment strategies and determine biomarkers that can identify good – and bad – candidates for different treatments.

## 7 Host factors governing TAMs

### 7.1 Diet and natural compounds

Evidence supporting the role of a healthy diet in cancer prevention and treatment is ever-increasing (187, 188). While natural compounds are known to have a profound role in regulating EMT in cancer cells (189, 190), numerous recent studies have also shed light on how dietary agents and natural compounds target tumor-associated macrophages.

#### 7.1.1 Antioxidants

Perhaps the most well-known dietary anti-cancer agents are antioxidants – compounds that neutralize free radicals that would otherwise damage DNA and other cellular structures and lead to carcinogenesis. Foods high in antioxidants include berries, fruits, vegetables, walnuts, and pecans (191). Recently, Latronico et al. demonstrated that dietary antioxidants act on macrophages and inhibit the expression and activity of macrophage-derived MMP-2 and MMP-9, which have pro-tumor ECM remodeling activity (see Section 3.2) (192). Macrophages mediate the production of reactive oxygen species (ROS) in the TME (193), and there is an established relationship between ROS and MMP production (194, 195). The authors posit that dietary antioxidants prevent MMP production by removing ROS in the microenvironment (192).

Propolis, a natural resin produced by honeybees, also has antioxidative properties (196–198). It is widely used as a natural additive in both ingestible (i.e. capsules, throat lozenges, food) and topical (i.e. lotions, cosmetics) products. Propolis induces the depolarization and repolarization of M2 macrophages to M0- and M1-like states, respectively. M2-polarized macrophages treated with propolis also express decreased levels of IL-8, IL-10, CCL2, VEGF, and MMP-9 (199). Consistent with this shift of M2 to M1 macrophages, propolis significantly decreases EMT and tumor cell migration and invasion (199).

For centuries, cloves have been used not only as a spice, but also as an herbal remedy due to their antimicrobial and antioxidative properties (200). Kumatakenin, a flavonoid isolated from cloves, has recently shown significant anti-cancer effects by acting on both tumor cells and tumor-associated macrophages (201). In addition to inducing apoptosis in human ovarian cancer cells, kumatakenin reduces tumor cell expression of CCL2 and CCL5 – both implicated in macrophage recruitment, cancer progression and metastasis (201–203). Kumatakenin also prevents M2 polarization and macrophage expression of IL-10, VEGF, MMP-2, and MMP-9 (201).

Together, these studies implicate antioxidants in reducing macrophage-mediated pro-tumoral effects including immunosuppression, angiogenesis, and pro-tumoral ECM remodeling.

#### 7.1.2 Vitamin D and omega-3 poly-unsaturated fatty acids

Vitamin D is a steroid hormone precursor that can come from the diet or be endogenously synthesized in the skin upon exposure to UV radiation (204). Vitamin D exerts its effects by binding to the vitamin D receptor and is primarily responsible for regulating calcium and phosphate levels in the body (205, 206). Though there is no clear consensus on the impact of vitamin D on cancer, a recent study showed that vitamin D may exert anti-cancer effects through macrophages. *In vitro* studies showed that vitamin D reverses M2 polarization, decreases macrophage secretion of TGF-β1 and MMP-9, and reduces the macrophage-induced proliferation and migration of ovarian cancer cells (207).

Omega-3 poly-unsaturated fatty acids (ω-3 PUFAs) – long lauded for their anti-inflammatory properties – also exhibit anti-cancer effects on tumor-associated macrophages (208). Studies on mouse models of castrate resistant prostate cancer show that a diet rich in ω-3 (vs. ω-6) PUFAs significantly delays tumor progression, decreases M2 polarization, and increases M1 polarization and infiltration of CD4+ T cells. M2 macrophages from tumors in mice fed a high ω-3 diet also show a significant decrease in MMP-9 and VEGF expression (208).

Together, these studies have begun to reveal the mechanisms by which a healthy diet can induce anti-cancer changes in macrophages and the TME more broadly.

### 7.2 Obesity

Obesity is a fast-growing global health crisis that is associated with an increased risk of cancer, as well as general morbidity and mortality (209, 210). In fact, women with obesity who are diagnosed with breast cancer have an increased risk of distant metastasis and are less likely to respond to some cancer treatments (211–214). Unsurprisingly, adipose tissue macrophages are thought to play a key role in creating a pro-tumorigenic microenvironment (86). CCL2 expression is significantly increased in the adipose tissue of obese compared to lean mice (215) which leads to the accumulation of macrophages. Indeed, it is estimated that macrophages make up <10% of cells in the adipose tissue of lean individuals and nearly 40% of cells in the adipose tissue of obese individuals (216). These recruited macrophages surround dead and dying adipocytes, forming crown-like structures (CLS) that are characteristic of adipose tissue inflammation (217). Overweight and obese patients (BMI ≥ 25 kg/m^2^) with breast cancer are more likely to have CLS compared to patients at a healthy weight (BMI < 25 kg/m^2^), and BMI ≥ 25 kg/m^2^ is associated with a shift in CLS macrophage phenotype that may be indicative of metabolic dysfunction and poor treatment outcomes under some conditions (218). These macrophages also interact with pre-adipocytes and prevent their differentiation, instead causing them to take on a fibroblastic phenotype and enhance the synthesis and deposition of ECM components (219, 220). This indicates that macrophages cause increased ECM density in obese adipose tissue, including in the breast where ECM density is a significant risk factor for cancer (72, 221). Further implicating adipose tissue macrophages in breast tumor development, a study on transgenic mice that overexpress CCL2 in the mammary epithelium showed that CCL2 overexpression causes increased macrophage density, stromal density, and ECM crosslinking enzyme LOX compared to non-transgenic controls (222). CCL2-overexpressing mice also had an increased susceptibility to DMBA-induced mammary tumors, demonstrating a relationship between macrophages, ECM density, and cancer risk (222).

Though much is still unknown about how obesity shapes cancer development, these studies suggest that obesity promotes macrophage-mediated ECM stiffening that is known to support tumorigenesis (see section 3.1).

### 7.3 Race

There are widespread racial disparities in the diagnosis, treatment, and outcome of cancer patients (223–226). While some of these disparities are due to systemic racism in the medical field, some studies identify biological differences that could be contributing to this effect (227). Indeed, Black men and women with prostate and breast cancer, respectively, have significantly worse outcomes than their white counterparts even after socioeconomic and other mediating factors are accounted for (228, 229). We previously evaluated distant recurrence-free survival (DRFS) in breast cancer patients following neoadjuvant (NAC) versus adjuvant (AC) chemotherapy and found that treatment type had no impact on DRFS for white women (230). However, Black women had significantly worse DRFS when treated with NAC (230). Differences in TAMs may contribute to this. Black breast cancer patients have significantly increased macrophage infiltration and M2 polarization compared to white patients, and this is prognostic of progression-free survival (231, 232). Black, compared to white women treated with neoadjuvant chemotherapy for ER+/HER2- breast cancer also have a higher TMEM doorway score and macrophage density in the residual tumor tissue, which may contribute to poorer outcomes (223). In summary, racial disparities in cancer development and outcome may be mediated in part by macrophage infiltration and activity.

## 8 Conclusion

**Figure 3:**
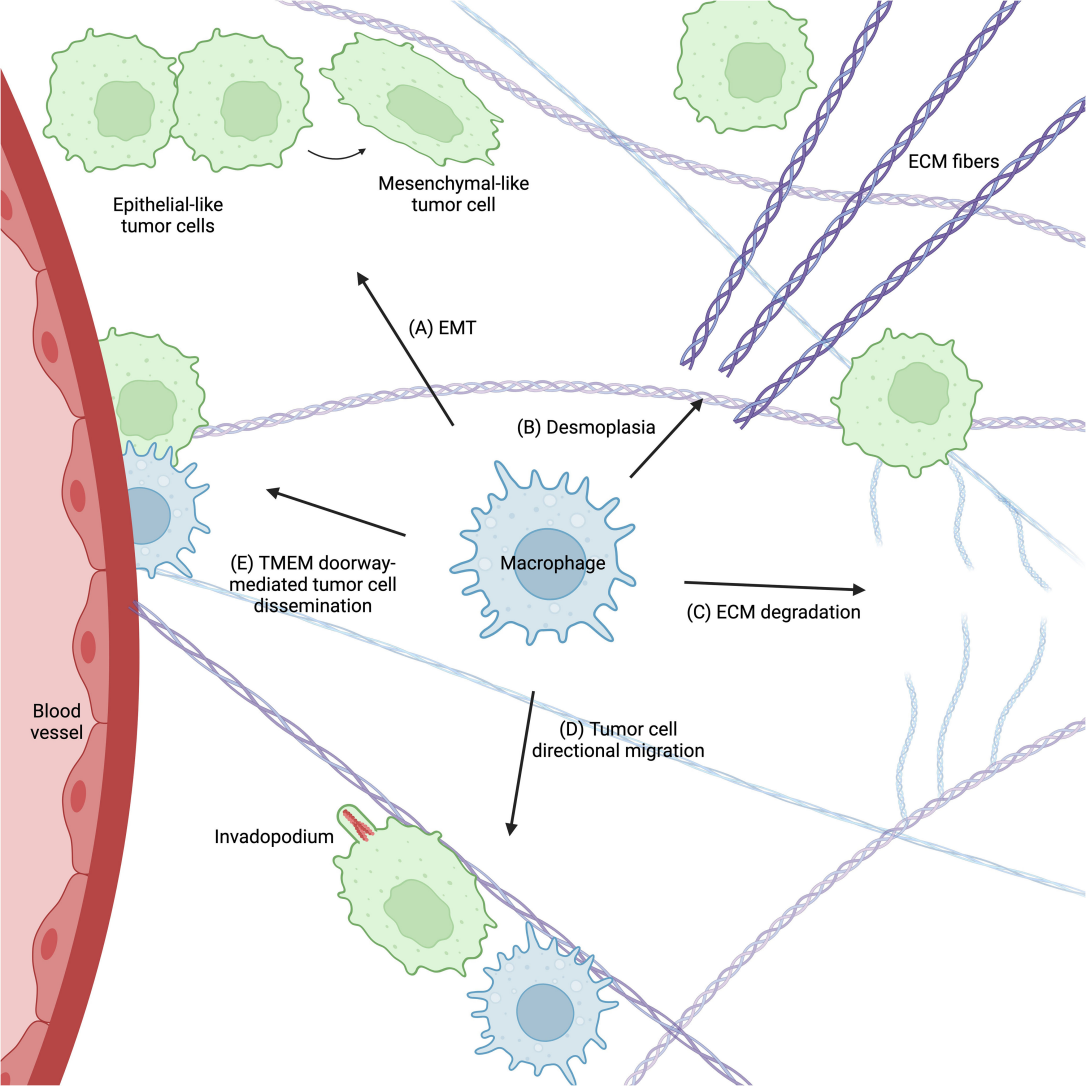
Macrophages support tumor cell migration and metastasis in many ways. **(A)** Macrophages promote EMT in tumor cells, which confers a migratory phenotype. They regulate ECM remodeling by enhancing both ECM stiffness (desmoplasia) **(B)** and degradation **(C)**, which supports tumor cell migration and metastasis. Macrophages also provide signals that promote tumor cell invadopodium formation and directional migration towards blood vessels **(D)**. Finally, macrophages participate in TMEM doorway-mediated tumor cell intravasation **(E)**, which allows tumor cells to disseminate throughout the body. Figure created with BioRender.com.

The diversity and complexity of tumor-associated macrophages leaves their functions highly context-dependent and variable. Despite this complexity, a consensus is emerging in the literature that tumor-associated macrophages support tumor cell migration and metastasis in many ways (**Figure 3**). TAMs confer migratory abilities in tumor cells by activating EMT. They remodel the ECM to facilitate tumor cell migration and provide ligands to promote invadopodium formation and chemotaxis. Finally, TAMs participate in the signaling cascade that opens TMEM doorways and allows tumor cells to intravasate and disseminate.

The plastic nature of TAMs means that their phenotypes and functions can dramatically change in response to environmental factors, including controllable factors such as chemotherapy, diet, and obesity and immutable factors such as race. Future research elucidating just how these factors play a role in macrophage function and cancer progression are crucial for refining treatment strategies.

## References

[1] Fidler IJ. The pathogenesis of cancer metastasis: the ‘seed and soil’ hypothesis revisited. Nat Rev Cancer. (2003) 3:453–8. doi: 10.1038/nrc1098 12778135

[2] Barisas DAG Choi K. Extramedullary hematopoiesis in cancer. Exp Mol Med. (2024) 56:549–58. doi: 10.1038/s12276-024-01192-4 PMC10985111 38443597

[3] Shi C Pamer EG. Monocyte recruitment during infection and inflammation. Nat Rev Immunol. (2011) 11:762–74. doi: 10.1038/nri3070 PMC3947780 21984070

[4] Davies LC Rosas M Jenkins SJ Liao C-T Scurr MJ Brombacher F. Distinct bone marrow-derived and tissue-resident macrophage lineages proliferate at key stages during inflammation. Nat Commun. (2013) 4:1886. doi: 10.1038/ncomms2877 23695680 PMC3842019

[5] Mass E Nimmerjahn F Kierdorf K Schlitzer A. Tissue-specific macrophages: how they develop and choreograph tissue biology. Nat Rev Immunol. (2023) 23:563–79. doi: 10.1038/s41577-023-00848-y PMC10017071 36922638

[6] Jappinen N Felix I Lokka E Tyystjarvi S Pynttari A Lahtela T. Fetal-derived macrophages dominate in adult mammary glands. Nat Commun. (2019) 10:281. doi: 10.1038/s41467-018-08065-1 30655530 PMC6336770

[7] Ginhoux F Guilliams M. Tissue-resident macrophage ontogeny and homeostasis. Immunity. (2016) 44:439–49. doi: 10.1016/j.immuni.2016.02.024 26982352

[8] Zhu Y Herndon JM Sojka DK Kim K-W Knolhoff BL Zuo C. Tissue-resident macrophages in pancreatic ductal adenocarcinoma originate from embryonic hematopoiesis and promote tumor progression. Immunity. (2017) 47:597. doi: 10.1016/j.immuni.2017.08.018 28930665 PMC5664180

[9] Hambardzumyan D Gutmann DH Kettenmann H. The role of microglia and macrophages in glioma maintenance and progression. Nat Neurosci. (2016) 19:20–7. doi: 10.1038/nn.4185 PMC4876023 26713745

[10] Bowman RL Klemm F Akkari L Pyonteck SM Sevenich L Quail DF. Macrophage ontogeny underlies differences in tumor-specific education in brain Malignancies. Cell Rep. (2016) 17:2445–59. doi: 10.1016/j.celrep.2016.10.052 PMC5450644 27840052

[11] Strizova Z Benesova I Bartolini R Novysedlak R Cecrdlova E Foley LK. M1/M2 macrophages and their overlaps - myth or reality? Clin Sci (Lond). (2023) 137:1067–93. doi: 10.1042/CS20220531 PMC10407193 37530555

[12] Wang H Wang X Li X Fan Y Li G Guo C. CD68(+)HLA-DR(+) M1-like macrophages promote motility of HCC cells via NF-κB/FAK pathway. Cancer Lett. (2014) 345:91–9. doi: 10.1016/j.canlet.2013.11.013 24333724

[13] Saleh R Taha RZ Sasidharan Nair V Toor SM Alajez NM Elkord E. Transcriptomic profiling of circulating HLA-DR(-) myeloid cells, compared with HLA-DR(+) myeloid antigen-presenting cells. Immunol Invest. (2021) 50:952–63. doi: 10.1080/08820139.2020.1795875 32727251

[14] Thiel M Wolfs MJ Bauer S Wenning AS Burckhart T Schwarz EC. Efficiency of T-cell costimulation by CD80 and CD86 cross-linking correlates with calcium entry. Immunology. (2010) 129:28–40. doi: 10.1111/j.1365-2567.2009.03155.x 19824921 PMC2807484

[15] Boutilier AJ Elsawa SF. Macrophage polarization states in the tumor microenvironment. Int J Mol Sci. (2021) 22:1-21. doi: 10.3390/ijms22136995 PMC8268869 34209703

[16] Garrido-Martin EM Mellows TWP Clarke J Ganesan A-P Wood O Cazaly A. M1(hot) tumor-associated macrophages boost tissue-resident memory T cells infiltration and survival in human lung cancer. J Immunother Cancer. (2020) 8:1-18. doi: 10.1136/jitc-2020-000778 PMC7375465 32699181

[17] Yan C Li K Meng F Chen L Zhao J Zhang Z. Integrated immunogenomic analysis of single-cell and bulk tissue transcriptome profiling unravels a macrophage activation paradigm associated with immunologically and clinically distinct behaviors in ovarian cancer. J Adv Res. (2023) 44:149–60. doi: 10.1016/j.jare.2022.04.006 PMC9936412 36725186

[18] Schaer CA Schoedon G Imhof A Kurrer MO Schaer DJ. Constitutive endocytosis of CD163 mediates hemoglobin-heme uptake and determines the noninflammatory and protective transcriptional response of macrophages to hemoglobin. Circ Res. (2006) 99:943–50. doi: 10.1161/01.RES.0000247067.34173.1b 17008602

[19] Azad AK Rajaram MV Schlesinger LS. Exploitation of the macrophage mannose receptor (CD206) in infectious disease diagnostics and therapeutics. J Cytol Mol Biol. (2014) 1:1-5. doi: 10.13188/2325-4653.1000003 PMC3963702 24672807

[20] Dan H Liu S Liu J Liu D Yin F Wei Z. RACK1 promotes cancer progression by increasing the M2/M1 macrophage ratio via the NF-κB pathway in oral squamous cell carcinoma. Mol Oncol. (2020) 14:795–807. doi: 10.1002/1878-0261.12644 31997535 PMC7138402

[21] Chen S Lu K Hou Y You Z Shu C Wei X. YY1 complex in M2 macrophage promotes prostate cancer progression by upregulating IL-6. J Immunother Cancer. (2023) 11:1-21. doi: 10.1136/jitc-2022-006020 37094986 PMC10152059

[22] Hu X Ma Z Xu B Li S Yao Z Liang B. Glutamine metabolic microenvironment drives M2 macrophage polarization to mediate trastuzumab resistance in HER2-positive gastric cancer. Cancer Commun (Lond). (2023) 43:909–37. doi: 10.1002/cac2.12459 PMC10397568 37434399

[23] Jiang H Wei H Wang H Wang Z Li J Ou Y. Zeb1-induced metabolic reprogramming of glycolysis is essential for macrophage polarization in breast cancer. Cell Death Dis. (2022) 13:206. doi: 10.1038/s41419-022-04632-z 35246504 PMC8897397

[24] Fujiwara T Fukushi J-i Yamamoto S Matsumoto Y Setsu N Oda Y. Macrophage infiltration predicts a poor prognosis for human ewing sarcoma. Am J Pathol. (2011) 179:1157–70. doi: 10.1016/j.ajpath.2011.05.034 PMC3157220 21771572

[25] Hirayama S Ishii G Nagai K Ono S Kojima M Yamauchi C. Prognostic impact of CD204-positive macrophages in lung squamous cell carcinoma: possible contribution of Cd204-positive macrophages to the tumor-promoting microenvironment. J Thorac Oncol. (2012) 7:1790–7. doi: 10.1097/JTO.0b013e3182745968 23154550

[26] Zhang H Wang X Shen Z Xu J Qin J Sun Y. Infiltration of diametrically polarized macrophages predicts overall survival of patients with gastric cancer after surgical resection. Gastric Cancer. (2015) 18:740–50. doi: 10.1007/s10120-014-0422-7 25231913

[27] McComb S Thiriot A Akache B Krishnan L Stark F. Introduction to the immune system. Methods Mol Biol. (2019) 2024:1–24. doi: 10.1007/978-1-4939-9597-4_1 31364040

[28] Davis MJ Tsang TM Qiu Y Dayrit JK Freij JB Huffnagle GB. Macrophage M1/M2 polarization dynamically adapts to changes in cytokine microenvironments in Cryptococcus neoformans infection. mBio. (2013) 4:e00264–13. doi: 10.1128/mBio.00264-13 PMC3684832 23781069

[29] Gao C-H Dong H-L Tai L Gao X-M. Lactoferrin-containing immunocomplexes drive the conversion of human macrophages from M2- into M1-like phenotype. Front Immunol. (2018) 9:37. doi: 10.3389/fimmu.2018.00037 29410669 PMC5787126

[30] Cheng H Sun L Shen D Ren A Ma F Tai G. Beta-1,6 glucan converts tumor-associated macrophages into an M1-like phenotype. Carbohydr Polym. (2020) 247:116715. doi: 10.1016/j.carbpol.2020.116715 32829842

[31] Wang S Yang Y Ma P Huang H Tang Q Miao H. Landscape and perspectives of macrophage -targeted cancer therapy in clinical trials. Mol Ther Oncolytics. (2022) 24:799–813. doi: 10.1016/j.omto.2022.02.019 35317518 PMC8908037

[32] Anderson NR Minutolo NG Gill S Klichinsky M. Macrophage-based approaches for cancer immunotherapy. Cancer Res. (2021) 81:1201–8. doi: 10.1158/0008-5472.CAN-20-2990 33203697

[33] Kashfi K Kannikal J Nath N. Macrophage reprogramming and cancer therapeutics: role of iNOS-derived NO. Cells. (2021) 10:1-22. doi: 10.3390/cells10113194 PMC8624911 34831416

[34] Ngambenjawong C Gustafson HH Pun SH. Progress in tumor-associated macrophage (TAM)-targeted therapeutics. Adv Drug Delivery Rev. (2017) 114:206–21. doi: 10.1016/j.addr.2017.04.010 PMC5581987 28449873

[35] Liu L Chen G Gong S Huang R Fan C. Targeting tumor-associated macrophage: an adjuvant strategy for lung cancer therapy. Front Immunol. (2023) 14:1274547. doi: 10.3389/fimmu.2023.1274547 38022518 PMC10679371

[36] Cassetta L Kitamura T. Macrophage targeting: opening new possibilities for cancer immunotherapy. Immunology. (2018) 155:285–93. doi: 10.1111/imm.2018.155.issue-3 PMC6187207 29963704

[37] Lopez-Yrigoyen M Cassetta L Pollard JW. Macrophage targeting in cancer. Ann N Y Acad Sci. (2021) 1499:18–41. doi: 10.1111/nyas.v1499.1 32445205

[38] Brabletz T Kalluri R Nieto MA Weinberg RA. EMT in cancer. Nat Rev Cancer. (2018) 18:128–34. doi: 10.1038/nrc.2017.118 29326430

[39] Kalluri R Weinberg RA. The basics of epithelial-mesenchymal transition. J Clin Invest. (2009) 119:1420–8. doi: 10.1172/JCI39104 PMC2689101 19487818

[40] Mendonsa AM Na TY Gumbiner BM. E-cadherin in contact inhibition and cancer. Oncogene. (2018) 37:4769–80. doi: 10.1038/s41388-018-0304-2 PMC6119098 29780167

[41] Cano A Pérez-Moreno MA Rodrigo I Locascio A Blanco MJ del Barrio MG. The transcription factor snail controls epithelial-mesenchymal transitions by repressing E-cadherin expression. Nat Cell Biol. (2000) 2:76–83. doi: 10.1038/35000025 10655586

[42] Loh CY Chai JY Tang TF Wong WF Sethi G Shanmugam MK. The E-cadherin and N-cadherin switch in epithelial-to-mesenchymal transition: signaling, therapeutic implications, and challenges. Cells. (2019) 8:1-33. doi: 10.3390/cells8101118 PMC6830116 31547193

[43] Usman S Waseem NH Nguyen TKN Mohsin S Jamal A Teh MT. Vimentin is at the heart of epithelial mesenchymal transition (EMT) mediated metastasis. Cancers (Basel). (2021) 13:1-26. doi: 10.3390/cancers13194985 PMC8507690 34638469

[44] Pastushenko I Brisebarre A Sifrim A Fioramonti M Revenco T Boumahdi S. Identification of the tumour transition states occurring during EMT. Nature. (2018) 556:463–8. doi: 10.1038/s41586-018-0040-3 29670281

[45] Su S Liu Q Chen J Chen J Chen F He C. A positive feedback loop between mesenchymal-like cancer cells and macrophages is essential to breast cancer metastasis. Cancer Cell. (2014) 25:605–20. doi: 10.1016/j.ccr.2014.03.021 24823638

[46] Liu C-Y Xu J-Y Shi X-Y Huang W Ruan T-Y Xie P. M2-polarized tumor-associated macrophages promoted epithelial-mesenchymal transition in pancreatic cancer cells, partially through TLR4/IL-10 signaling pathway. Lab Invest. (2013) 93:844–54. doi: 10.1038/labinvest.2013.69 23752129

[47] Wei C Yang C Wang S Shi D Zhang C Lin X. Crosstalk between cancer cells and tumor associated macrophages is required for mesenchymal circulating tumor cell-mediated colorectal cancer metastasis. Mol Cancer. (2019) 18:64. doi: 10.1186/s12943-019-0976-4 30927925 PMC6441214

[48] Hao Y Baker D Ten Dijke P. TGF-β-mediated epithelial-mesenchymal transition and cancer metastasis. Int J Mol Sci. (2019) 20:1-11. doi: 10.3390/ijms20112767 PMC6600375 31195692

[49] Ling Z Yang X Chen X Xia J Cheng B Tao X. CCL2 promotes cell migration by inducing epithelial-mesenchymal transition in oral squamous cell carcinoma. J Oral Pathol Med. (2019) 48:477–82. doi: 10.1111/jop.2019.48.issue-6 31077446

[50] Rokavec M Öner MG Li H Jackstadt R Jiang L Lodygin D. IL-6R/STAT3/miR-34a feedback loop promotes EMT-mediated colorectal cancer invasion and metastasis. J Clin Invest. (2014) 124:1853–67. doi: 10.1172/JCI73531 PMC3973098 24642471

[51] Weng Y-S Tseng H-Y Chen Y-A Shen P-C Al Haq AT Chen L-M. MCT-1/miR-34a/IL-6/IL-6R signaling axis promotes EMT progression, cancer stemness and M2 macrophage polarization in triple-negative breast cancer. Mol Cancer. (2019) 18:42. doi: 10.1186/s12943-019-0988-0 30885232 PMC6421700

[52] Huang B Lang X Li X. The role of IL-6/JAK2/STAT3 signaling pathway in cancers. Front Oncol. (2022) 12:1023177. doi: 10.3389/fonc.2022.1023177 36591515 PMC9800921

[53] Jin W. Role of JAK/STAT3 signaling in the regulation of metastasis, the transition of cancer stem cells, and chemoresistance of cancer by epithelial-mesenchymal transition. Cells. (2020) 9:1-23. doi: 10.3390/cells9010217 PMC7017057 31952344

[54] Kim NH Kim HS Li X-Y Lee I Choi H-S Kang SE. A p53/miRNA-34 axis regulates Snail1-dependent cancer cell epithelial-mesenchymal transition. J Cell Biol. (2011) 195:417–33. doi: 10.1083/jcb.201103097 PMC3206336 22024162

[55] Siemens H Jackstadt R Hünten S Kaller M Menssen A Götz U. miR-34 and SNAIL form a double-negative feedback loop to regulate epithelial-mesenchymal transitions. Cell Cycle. (2011) 10:4256–71. doi: 10.4161/cc.10.24.18552 22134354

[56] Liu C Rokavec M Huang Z Hermeking H. Curcumin activates a ROS/KEAP1/NRF2/miR-34a/b/c cascade to suppress colorectal cancer metastasis. Cell Death Differ. (2023) 30:1771–85. doi: 10.1038/s41418-023-01178-1 PMC10307888 37210578

[57] Fu X-L Duan W Su C-Y Mao F-Y Lv Y-P Teng Y-S. Interleukin 6 induces M2 macrophage differentiation by STAT3 activation that correlates with gastric cancer progression. Cancer Immunol Immunother. (2017) 66:1597–608. doi: 10.1007/s00262-017-2052-5 PMC11028627 28828629

[58] Dehghankelishadi P Maritz MF Badiee P Thierry B. High density lipoprotein nanoparticle as delivery system for radio-sensitising miRNA: An investigation in 2D/3D head and neck cancer models. Int J Pharm. (2022) 617:121585. doi: 10.1016/j.ijpharm.2022.121585 35176332

[59] Shi S Han L Deng L Zhang Y Shen H Gong T. Dual drugs (microRNA-34a and paclitaxel)-loaded functional solid lipid nanoparticles for synergistic cancer cell suppression. J Control Release. (2014) 194:228–37. doi: 10.1016/j.jconrel.2014.09.005 25220161

[60] Hsu DS-S Wang H-J Tai S-K Chou C-H Hsieh C-H Chiu P-H. Acetylation of snail modulates the cytokinome of cancer cells to enhance the recruitment of macrophages. Cancer Cell. (2014) 26:534–48. doi: 10.1016/j.ccell.2014.09.002 25314079

[61] Ouyang P Li K Xu W Chen C Shi Y Tian Y. METTL3 recruiting M2-type immunosuppressed macrophages by targeting m6A-SNAIL-CXCL2 axis to promote colorectal cancer pulmonary metastasis. J Exp Clin Cancer Res. (2024) 43:111. doi: 10.1186/s13046-024-03035-6 38605400 PMC11007974

[62] Bao Z Zeng W Zhang D Wang L Deng X Lai J. SNAIL induces EMT and lung metastasis of tumours secreting CXCL2 to promote the invasion of M2-type immunosuppressed macrophages in colorectal cancer. Int J Biol Sci. (2022) 18:2867–81. doi: 10.7150/ijbs.66854 PMC9066124 35541899

[63] Yang C Dou R Wei C Liu K Shi D Zhang C. Tumor-derived exosomal microRNA-106b-5p activates EMT-cancer cell and M2-subtype TAM interaction to facilitate CRC metastasis. Mol Ther. (2021) 29:2088–107. doi: 10.1016/j.ymthe.2021.02.006 PMC8178444 33571679

[64] Hsieh C-H Tai S-K Yang M-H. Snail-overexpressing cancer cells promote M2-like polarization of tumor-associated macrophages by delivering miR-21-abundant exosomes. Neoplasia. (2018) 20:775–88. doi: 10.1016/j.neo.2018.06.004 PMC6031090 29981499

[65] Lu Y Han G Zhang Y Zhang L Li Z Wang Q. M2 macrophage-secreted exosomes promote metastasis and increase vascular permeability in hepatocellular carcinoma. Cell Communication and Signaling (2023) 21:1-16. doi: 10.21203/rs.3.rs-1225380/v1 37904170 PMC10614338

[66] Fedorova O Parfenyev S Daks A Shuvalov O Barlev NA. The role of PTEN in epithelial-mesenchymal transition. Cancers (Basel). (2022) 14:1-22. doi: 10.3390/cancers14153786 PMC9367281 35954450

[67] Egeblad M Rasch MG Weaver VM. Dynamic interplay between the collagen scaffold and tumor evolution. Curr Opin Cell Biol. (2010) 22:697–706. doi: 10.1016/j.ceb.2010.08.015 20822891 PMC2948601

[68] Aw Yong KM Sun Y Merajver SD Fu J. Mechanotransduction-induced reversible phenotypic switching in prostate cancer cells. Biophys J. (2017) 112:1236–45. doi: 10.1016/j.bpj.2017.02.012 PMC5376107 28355550

[69] Fattet L Jung H-Y Matsumoto MW Aubol BE Kumar A Adams JA. Matrix rigidity controls epithelial-mesenchymal plasticity and tumor metastasis via a mechanoresponsive EPHA2/LYN complex. Dev Cell. (2020) 54:302–16.e7. doi: 10.1016/j.devcel.2020.05.031 32574556 PMC7423770

[70] Llovet JM Kelley RK Villanueva A Singal AG Pikarsky E Roayaie S. Hepatocellular carcinoma. Nat Rev Dis Primers. (2021) 7:6. doi: 10.1038/s41572-020-00240-3 33479224 PMC13537433

[71] Ballester B Milara J Cortijo J. Idiopathic pulmonary fibrosis and lung cancer: mechanisms and molecular targets. Int J Mol Sci. (2019) 20:1-28. doi: 10.3390/ijms20030593 PMC6387034 30704051

[72] Bodewes FTH van Asselt AA Dorrius MD Greuter MJW de Bock GH. Mammographic breast density and the risk of breast cancer: A systematic review and meta-analysis. Breast. (2022) 66:62–8. doi: 10.1016/j.breast.2022.09.007 PMC9530665 36183671

[73] Akimoto N Väyrynen JP Zhao M Ugai T Fujiyoshi K Borowsky J. Desmoplastic reaction, immune cell response, and prognosis in colorectal cancer. Front Immunol. (2022) 13:840198. doi: 10.3389/fimmu.2022.840198 35392092 PMC8980356

[74] Wolf B Weydandt L Dornhöfer N Hiller GGR Höhn AK Nel I. Desmoplasia in cervical cancer is associated with a more aggressive tumor phenotype. Sci Rep. (2023) 13:18946. doi: 10.1038/s41598-023-46340-4 37919378 PMC10622496

[75] Provenzano PP Eliceiri KW Campbell JM Inman DR White JG Keely PJ. Collagen reorganization at the tumor-stromal interface facilitates local invasion. BMC Med. (2006) 4:38. doi: 10.1186/1741-7015-4-38 17190588 PMC1781458

[76] Levental KR Yu H Kass L Lakins JN Egeblad M Erler JT. Matrix crosslinking forces tumor progression by enhancing integrin signaling. Cell. (2009) 139:891–906. doi: 10.1016/j.cell.2009.10.027 19931152 PMC2788004

[77] Paszek MJ Zahir N Johnson KR Lakins JN Rozenberg GI Gefen A. Tensional homeostasis and the Malignant phenotype. Cancer Cell. (2005) 8:241–54. doi: 10.1016/j.ccr.2005.08.010 16169468

[78] Itoh Y Takehara Y Kawase T Terashima K Ohkawa Y Hirose Y. Feasibility of magnetic resonance elastography for the pancreas at 3T. J Magn Reson Imaging. (2016) 43:384–90. doi: 10.1002/jmri.24995 26149267

[79] Ghasemi H Mousavibahar SH Hashemnia M Karimi J Khodadadi I Mirzaei F. Tissue stiffness contributes to YAP activation in bladder cancer patients undergoing transurethral resection. Ann N Y Acad Sci. (2020) 1473:48–61. doi: 10.1111/nyas.v1473.1 32428277

[80] Mieulet V Garnier C Kieffer Y Guilbert T Nemati F Marangoni E. Stiffness increases with myofibroblast content and collagen density in mesenchymal high grade serous ovarian cancer. Sci Rep. (2021) 11:4219. doi: 10.1038/s41598-021-83685-0 33603134 PMC7892556

[81] Provenzano PP Inman DR Eliceiri KW Keely PJ. Matrix density-induced mechanoregulation of breast cell phenotype, signaling and gene expression through a FAK-ERK linkage. Oncogene. (2009) 28:4326–43. doi: 10.1038/onc.2009.299 PMC2795025 19826415

[82] Wei SC Fattet L Tsai JH Guo Y Pai VH Majeski HE. Matrix stiffness drives epithelial-mesenchymal transition and tumour metastasis through a TWIST1-G3BP2 mechanotransduction pathway. Nat Cell Biol. (2015) 17:678–88. doi: 10.1038/ncb3157 PMC4452027 25893917

[83] Dai J Qin L Chen Y Wang H Lin G Li X. Matrix stiffness regulates epithelial-mesenchymal transition via cytoskeletal remodeling and MRTF-A translocation in osteosarcoma cells. J Mech Behav BioMed Mater. (2019) 90:226–38. doi: 10.1016/j.jmbbm.2018.10.012 30384218

[84] Han W Chen S Yuan W Fan Q Tian J Wang X. Oriented collagen fibers direct tumor cell intravasation. Proc Natl Acad Sci U S A. (2016) 113:11208–13. doi: 10.1073/pnas.1610347113 PMC5056065 27663743

[85] Riching KM Cox BL Salick MR Pehlke C Riching AS Ponik SM. 3D collagen alignment limits protrusions to enhance breast cancer cell persistence. Biophys J. (2014) 107:2546–58. doi: 10.1016/j.bpj.2014.10.035 PMC4255204 25468334

[86] Kuziel G Moore BN Arendt LM. Obesity and fibrosis: setting the stage for breast cancer. Cancers (Basel). (2023) 15:1-22. doi: 10.3390/cancers15112929 PMC10252103 37296891

[87] LaRue MM Parker S Puccini J Cammer M Kimmelman AC Bar-Sagi D. Metabolic reprogramming of tumor-associated macrophages by collagen turnover promotes fibrosis in pancreatic cancer. Proc Natl Acad Sci U S A. (2022) 119:e2119168119. doi: 10.1073/pnas.2119168119 35412885 PMC9169723

[88] Ishihara S Haga H. Matrix stiffness contributes to cancer progression by regulating transcription factors. Cancers (Basel). (2022) 14:1-17. doi: 10.3390/cancers14041049 PMC8870363 35205794

[89] Alonso-Nocelo M Ruiz-Cañas L Sancho P Görgülü K Alcalá S Pedrero C. Macrophages direct cancer cells through a LOXL2-mediated metastatic cascade in pancreatic ductal adenocarcinoma. Gut. (2023) 72:345–59. doi: 10.1136/gutjnl-2021-325564 PMC9872246 35428659

[90] Xing X Wang Y Zhang X Gao X Li M Wu S. Matrix stiffness-mediated effects on macrophages polarization and their LOXL2 expression. FEBS J. (2021) 288:3465–77. doi: 10.1111/febs.v288.11 32964626

[91] Larsen AMH Kuczek DE Kalvisa A Siersbæk MS Thorseth M-L Johansen AZ. Collagen density modulates the immunosuppressive functions of macrophages. J Immunol. (2020) 205:1461–72. doi: 10.4049/jimmunol.1900789 32839214

[92] Kapoor C Vaidya S Wadhwan V Kaur G Pathak A. Seesaw of matrix metalloproteinases (MMPs). J Cancer Res Ther. (2016) 12:28–35. doi: 10.4103/0973-1482.157337 27072206

[93] Zeng Y Gao M Lin D Du G Cai Y. Prognostic and immunological roles of MMP-9 in pan-cancer. BioMed Res Int. (2022) 2022:2592962. doi: 10.1155/2022/2592962 35178444 PMC8844435

[94] Jia Z-H Jia Y Guo F-J Chen J Zhang X-W Cui M-H. Phosphorylation of STAT3 at Tyr705 regulates MMP-9 production in epithelial ovarian cancer. PloS One. (2017) 12:e0183622. doi: 10.1371/journal.pone.0183622 28859117 PMC5578655

[95] Liu X Lv Z Zou J Liu X Ma J Sun C. Elevated AEG-1 expression in macrophages promotes hypopharyngeal cancer invasion through the STAT3-MMP-9 signaling pathway. Oncotarget. (2016) 7:77244–56. doi: 10.18632/oncotarget.12886 PMC5363584 27793010

[96] Zhang F Wang Z Fan Y Xu Q Ji W Tian R. Elevated STAT3 signaling-mediated upregulation of MMP-2/9 confers enhanced invasion ability in multidrug-resistant breast cancer cells. Int J Mol Sci. (2015) 16:24772–90. doi: 10.3390/ijms161024772 PMC4632776 26501276

[97] Zhu X Liang R Lan T Ding D Huang S Shao J. Tumor-associated macrophage-specific CD155 contributes to M2-phenotype transition, immunosuppression, and tumor progression in colorectal cancer. J Immunother Cancer. (2022) 10:1-14. doi: 10.1136/jitc-2021-004219 PMC9476138 36104099

[98] Vinnakota K Zhang Y Selvanesan BC Topi G Salim T Sand-Dejmek J. M2-like macrophages induce colon cancer cell invasion via matrix metalloproteinases. J Cell Physiol. (2017) 232:3468–80. doi: 10.1002/jcp.v232.12 28098359

[99] Cho SJ Jeong BY Song YS Park CG Cho DY Lee HY. STAT3 mediates RCP-induced cancer cell invasion through the NF-κB/Slug/MT1-MMP signaling cascade. Arch Pharm Res. (2022) 45:460–74. doi: 10.1007/s12272-022-01396-0 35809175

[100] Augoff K Hryniewicz-Jankowska A Tabola R Stach K. MMP9: A tough target for targeted therapy for cancer. Cancers (Basel). (2022) 14:1-28. doi: 10.3390/cancers14071847 PMC8998077 35406619

[101] Li X Bao C Ma Z Xu B Ying X Liu X. Perfluorooctanoic acid stimulates ovarian cancer cell migration, invasion via ERK/NF-κB/MMP-2/-9 pathway. Toxicol Lett. (2018) 294:44–50. doi: 10.1016/j.toxlet.2018.05.009 29753068

[102] Wen J Yin P Su Y Gao F Wu Y Zhang W. Knockdown of HMGB1 inhibits the crosstalk between oral squamous cell carcinoma cells and tumor-associated macrophages. Int Immunopharmacol. (2023) 119:110259. doi: 10.1016/j.intimp.2023.110259 37141670

[103] Yamanaka N Morisaki T Nakashima H Tasaki A Kubo M Kuga H. Interleukin 1beta enhances invasive ability of gastric carcinoma through nuclear factor-kappaB activation. Clin Cancer Res. (2004) 10:1853–9. doi: 10.1158/1078-0432.CCR-03-0300 15014040

[104] Kang SU Cho SY Jeong H Han J Chae HY Yang H. Matrix metalloproteinase 11 (MMP11) in macrophages promotes the migration of HER2-positive breast cancer cells and monocyte recruitment through CCL2-CCR2 signaling. Lab Invest. (2022) 102:376–90. doi: 10.1038/s41374-021-00699-y 34775491

[105] Tian K Du G Wang X Wu X Li L Liu W. MMP-9 secreted by M2-type macrophages promotes Wilms’ tumour metastasis through the PI3K/AKT pathway. Mol Biol Rep. (2022) 49:3469–80. doi: 10.1007/s11033-022-07184-9 35107742

[106] Liu L Ye Y Zhu X. MMP-9 secreted by tumor associated macrophages promoted gastric cancer metastasis through a PI3K/AKT/Snail pathway. BioMed Pharmacother. (2019) 117:109096. doi: 10.1016/j.biopha.2019.109096 31202170

[107] Wyckoff JB Wang Y Lin EY J-f Li Goswami S ER S. Direct visualization of macrophage-assisted tumor cell intravasation in mammary tumors. Cancer Res. (2007) 67:2649–56. doi: 10.1158/0008-5472.CAN-06-1823 17363585

[108] Goswami S Sahai E Wyckoff JB Cammer M Cox D Pixley FJ. Macrophages promote the invasion of breast carcinoma cells via a colony-stimulating factor-1/epidermal growth factor paracrine loop. Cancer Res. (2005) 65:5278–83. doi: 10.1158/0008-5472.CAN-04-1853 15958574

[109] Roussos ET Balsamo M Alford SK Wyckoff JB Gligorijevic B Wang Y. Mena invasive (MenaINV) promotes multicellular streaming motility and transendothelial migration in a mouse model of breast cancer. J Cell Sci. (2011) 124:2120–31. doi: 10.1242/jcs.086231 PMC3113666 21670198

[110] Patsialou A Bravo-Cordero JJ Wang Y Entenberg D Liu H Clarke M. Intravital multiphoton imaging reveals multicellular streaming as a crucial component of in *vivo* cell migration in human breast tumors. Intravital. (2013) 2:e25294. doi: 10.4161/intv.25294 25013744 PMC3908591

[111] Smith HO Anderson PS Kuo DY Goldberg GL DeVictoria CL Boocock CA. The role of colony-stimulating factor 1 and its receptor in the etiopathogenesis of endometrial adenocarcinoma. Clin Cancer Res. (1995) 1:313–25. 9815987

[112] Yang L Wu Q Xu L Zhang W Zhu Y Liu H. Increased expression of colony stimulating factor-1 is a predictor of poor prognosis in patients with clear-cell renal cell carcinoma. BMC Cancer. (2015) 15:67. doi: 10.1186/s12885-015-1076-5 25886010 PMC4339479

[113] Mroczko B Groblewska M Wereszczyńska-Siemiatkowska U Okulczyk B Kedra B Łaszewicz W. Serum macrophage-colony stimulating factor levels in colorectal cancer patients correlate with lymph node metastasis and poor prognosis. Clin Chim Acta. (2007) 380:208–12. doi: 10.1016/j.cca.2007.02.037 17368603

[114] Klijn JG Look MP Portengen H Alexieva-Figusch J van Putten WL Foekens JA. The prognostic value of epidermal growth factor receptor (EGF-R) in primary breast cancer: results of a 10 year follow-up study. Breast Cancer Res Treat. (1994) 29:73–83. doi: 10.1007/BF00666183 8018964

[115] Lo H-W Xia W Wei Y Ali-Seyed M Huang S-F Hung M-C. Novel prognostic value of nuclear epidermal growth factor receptor in breast cancer. Cancer Res. (2005) 65:338–48. doi: 10.1158/0008-5472.338.65.1 15665312

[116] Li C Iida M Dunn EF Ghia AJ Wheeler DL. Nuclear EGFR contributes to acquired resistance to cetuximab. Oncogene. (2009) 28:3801–13. doi: 10.1038/onc.2009.234 PMC2900381 19684613

[117] Traynor AM Weigel TL Oettel KR Yang DT Zhang C Kim K. Nuclear EGFR protein expression predicts poor survival in early stage non-small cell lung cancer. Lung Cancer. (2013) 81:138–41. doi: 10.1016/j.lungcan.2013.03.020 PMC3679338 23628526

[118] Yamaguchi H Lorenz M Kempiak S Sarmiento C Coniglio S Symons M. Molecular mechanisms of invadopodium formation: the role of the N-WASP-Arp2/3 complex pathway and cofilin. J Cell Biol. (2005) 168:441–52. doi: 10.1083/jcb.200407076 PMC2171731 15684033

[119] Leung E Xue A Wang Y Rougerie P Sharma VP Eddy R. Blood vessel endothelium-directed tumor cell streaming in breast tumors requires the HGF/C-Met signaling pathway. Oncogene. (2017) 36:2680–92. doi: 10.1038/onc.2016.421 PMC5426963 27893712

[120] Eddy RJ Weidmann MD Sharma VP Condeelis JS. Tumor cell invadopodia: invasive protrusions that orchestrate metastasis. Trends Cell Biol. (2017) 27:595–607. doi: 10.1016/j.tcb.2017.03.003 28412099 PMC5524604

[121] Sibony-Benyamini H Gil-Henn H. Invadopodia: the leading force. Eur J Cell Biol. (2012) 91:896–901. doi: 10.1016/j.ejcb.2012.04.001 22633185

[122] Jeannot P Besson A. Cortactin function in invadopodia. Small GTPases. (2020) 11:256–70. doi: 10.1080/21541248.2017.1405773 PMC7549685 29172953

[123] Mader CC Oser M Magalhaes MA Bravo-Cordero JJ Condeelis J Koleske AJ. An EGFR-Src-Arg-cortactin pathway mediates functional maturation of invadopodia and breast cancer cell invasion. Cancer Res. (2011) 71:1730–41. doi: 10.1158/0008-5472.CAN-10-1432 PMC3057139 21257711

[124] Gertler FB Niebuhr K Reinhard M Wehland J Soriano P. Mena, a relative of VASP and Drosophila Enabled, is implicated in the control of microfilament dynamics. Cell. (1996) 87:227–39. doi: 10.1016/S0092-8674(00)81341-0 8861907

[125] Bear JE Svitkina TM Krause M Schafer DA Loureiro JJ Strasser GA. Antagonism between Ena/VASP proteins and actin filament capping regulates fibroblast motility. Cell. (2002) 109:509–21. doi: 10.1016/S0092-8674(02)00731-6 12086607

[126] Philippar U Roussos ET Oser M Yamaguchi H Kim H-D Giampieri S. A Mena invasion isoform potentiates EGF-induced carcinoma cell invasion and metastasis. Dev Cell. (2008) 15:813–28. doi: 10.1016/j.devcel.2008.09.003 PMC2637261 19081071

[127] Hughes SK Oudin MJ Tadros J Neil J Del Rosario A Joughin BA. PTP1B-dependent regulation of receptor tyrosine kinase signaling by the actin-binding protein Mena. Mol Biol Cell. (2015) 26:3867–78. doi: 10.1091/mbc.E15-06-0442 PMC4626070 26337385

[128] Weidmann MD Surve CR Eddy RJ Chen X Gertler FB Sharma VP. Mena(INV) dysregulates cortactin phosphorylation to promote invadopodium maturation. Sci Rep. (2016) 6:36142. doi: 10.1038/srep36142 27824079 PMC5099927

[129] Pignatelli J Bravo-Cordero JJ Roh-Johnson M Gandhi SJ Wang Y Chen X. Macrophage-dependent tumor cell transendothelial migration is mediated by Notch1/Mena(INV)-initiated invadopodium formation. Sci Rep. (2016) 6:37874. doi: 10.1038/srep37874 27901093 PMC5129016

[130] Duran CL Karagiannis GS Chen X Sharma VP Entenberg D Condeelis JS. Cooperative NF-κB and Notch1 signaling promotes macrophage-mediated MenaINV expression in breast cancer. Breast Cancer Res. (2023) 25:37. doi: 10.1186/s13058-023-01628-1 37024946 PMC10080980

[131] Goswami S Philippar U Sun D Patsialou A Avraham J Wang W. Identification of invasion specific splice variants of the cytoskeletal protein Mena present in mammary tumor cells during invasion in vivo. Clin Exp Metastasis. (2009) 26:153–9. doi: 10.1007/s10585-008-9225-8 PMC3042857 18985426

[132] Harney AS Arwert EN Entenberg D Wang Y Guo P Qian B-Z. Real-time imaging reveals local, transient vascular permeability, and tumor cell intravasation stimulated by TIE2hi macrophage-derived VEGFA. Cancer Discovery. (2015) 5:932–43. doi: 10.1158/2159-8290.CD-15-0012 PMC4560669 26269515

[133] Karagiannis GS Pastoriza JM Wang Y Harney AS Entenberg D Pignatelli J. Neoadjuvant chemotherapy induces breast cancer metastasis through a TMEM-mediated mechanism. Sci Transl Med. (2017) 9:1-30. doi: 10.1126/scitranslmed.aan0026 PMC5592784 28679654

[134] Oktay MH Jones JG. TMEM: a novel breast cancer dissemination marker for the assessment of metastatic risk. biomark Med. (2015) 9:81–4. doi: 10.2217/bmm.14.104 25689896

[135] Roussos ET Goswami S Balsamo M Wang Y Stobezki R Adler E. Mena invasive (Mena(INV)) and Mena11a isoforms play distinct roles in breast cancer cell cohesion and association with TMEM. Clin Exp Metastasis. (2011) 28:515–27. doi: 10.1007/s10585-011-9388-6 PMC3459587 21484349

[136] Rohan TE Xue X Lin H-M D’Alfonso TM Ginter PS Oktay MH. Tumor microenvironment of metastasis and risk of distant metastasis of breast cancer. J Natl Cancer Inst. (2014) 106:1-11. doi: 10.1093/jnci/dju136 PMC4133559 24895374

[137] Robinson BD Sica GL Liu Y-F Rohan TE Gertler FB Condeelis JS. Tumor microenvironment of metastasis in human breast carcinoma: a potential prognostic marker linked to hematogenous dissemination. Clin Cancer Res. (2009) 15:2433–41. doi: 10.1158/1078-0432.CCR-08-2179 PMC3156570 19318480

[138] Sparano JA Gray R Oktay MH Entenberg D Rohan T Xue X. A metastasis biomarker (MetaSite Breast™ Score) is associated with distant recurrence in hormone receptor-positive, HER2-negative early-stage breast cancer. NPJ Breast Cancer. (2017) 3:42. doi: 10.1038/s41523-017-0043-5 29138761 PMC5678158

[139] Arwert EN Harney AS Entenberg D Wang Y Sahai E Pollard JW. A unidirectional transition from migratory to perivascular macrophage is required for tumor cell intravasation. Cell Rep. (2018) 23:1239–48. doi: 10.1016/j.celrep.2018.04.007 PMC5946803 29719241

[140] Roh-Johnson M Bravo-Cordero JJ Patsialou A Sharma VP Guo P Liu H. Macrophage contact induces RhoA GTPase signaling to trigger tumor cell intravasation. Oncogene. (2014) 33:4203–12. doi: 10.1038/onc.2013.377 PMC3962803 24056963

[141] Gil-Henn H Patsialou A Wang Y Warren MS Condeelis JS Koleske AJ. Arg/Abl2 promotes invasion and attenuates proliferation of breast cancer in vivo. Oncogene. (2013) 32:2622–30. doi: 10.1038/onc.2012.284 PMC3473103 22777352

[142] Gligorijevic B Wyckoff J Yamaguchi H Wang Y Roussos ET. Condeelis J. N-WASP-mediated invadopodium formation is involved in intravasation and lung metastasis of mammary tumors. J Cell Sci. (2012) 125:724–34. doi: 10.1242/jcs.092726 PMC3367832 22389406

[143] Blouw B Seals DF Pass I Diaz B Courtneidge SA. A role for the podosome/invadopodia scaffold protein Tks5 in tumor growth in *vivo* . Eur J Cell Biol. (2008) 87:555–67. doi: 10.1016/j.ejcb.2008.02.008 PMC2629379 18417249

[144] Sakurai-Yageta M Recchi C Le Dez G Sibarita J-B Daviet L Camonis J. The interaction of IQGAP1 with the exocyst complex is required for tumor cell invasion downstream of Cdc42 and RhoA. J Cell Biol. (2008) 181:985–98. doi: 10.1083/jcb.200709076 PMC2426946 18541705

[145] Harney AS Karagiannis GS Pignatelli J Smith BD Kadioglu E Wise SC. The selective tie2 inhibitor rebastinib blocks recruitment and function of tie2(Hi) macrophages in breast cancer and pancreatic neuroendocrine tumors. Mol Cancer Ther. (2017) 16:2486–501. doi: 10.1158/1535-7163.MCT-17-0241 PMC5669998 28838996

[146] Tilsed CM Fisher SA Nowak AK Lake RA Lesterhuis WJ. Cancer chemotherapy: insights into cellular and tumor microenvironmental mechanisms of action. Front Oncol. (2022) 12:960317. doi: 10.3389/fonc.2022.960317 35965519 PMC9372369

[147] Perera PY Vogel SN Detore GR Haziot A Goyert SM. CD14-dependent and CD14-independent signaling pathways in murine macrophages from normal and CD14 knockout mice stimulated with lipopolysaccharide or taxol. J Immunol. (1997) 158:4422–9. doi: 10.4049/jimmunol.158.9.4422 9127007

[148] Wanderley CW Colón DF Luiz JPM Oliveira FF Viacava PR Leite CA. Paclitaxel reduces tumor growth by reprogramming tumor-associated macrophages to an M1 profile in a TLR4-dependent manner. Cancer Res. (2018) 78:5891–900. doi: 10.1158/0008-5472.CAN-17-3480 30104241

[149] Cullis J Siolas D Avanzi A Barui S Maitra A Bar-Sagi D. Macropinocytosis of nab-paclitaxel drives macrophage activation in pancreatic cancer. Cancer Immunol Res. (2017) 5:182–90. doi: 10.1158/2326-6066.CIR-16-0125 PMC5570452 28108630

[150] Mullins DW Burger CJ Elgert KD. Paclitaxel enhances macrophage IL-12 production in tumor-bearing hosts through nitric oxide. J Immunol. (1999) 162:6811–8. doi: 10.4049/jimmunol.162.11.6811 10352302

[151] Rajput S Volk-Draper LD Ran S. TLR4 is a novel determinant of the response to paclitaxel in breast cancer. Mol Cancer Ther. (2013) 12:1676–87. doi: 10.1158/1535-7163.MCT-12-1019 PMC3742631 23720768

[152] Li Y Adamek P Zhang H Tatsui CE Rhines LD Mrozkova P. The cancer chemotherapeutic paclitaxel increases human and rodent sensory neuron responses to TRPV1 by activation of TLR4. J Neurosci. (2015) 35:13487–500. doi: 10.1523/JNEUROSCI.1956-15.2015 PMC4588613 26424893

[153] Volk-Draper L Hall K Griggs C Rajput S Kohio P DeNardo D. Paclitaxel therapy promotes breast cancer metastasis in a TLR4-dependent manner. Cancer Res. (2014) 74:5421–34. doi: 10.1158/0008-5472.CAN-14-0067 PMC4185415 25274031

[154] Resman N Oblak A Gioannini TL Weiss JP Jerala R. Tetraacylated lipid A and paclitaxel-selective activation of TLR4/MD-2 conferred through hydrophobic interactions. J Immunol. (2014) 192:1887–95. doi: 10.4049/jimmunol.1302119 24420921

[155] Zimmer SM Liu J Clayton JL Stephens DS Snyder JP. Paclitaxel binding to human and murine MD-2. J Biol Chem. (2008) 283:27916–26. doi: 10.1074/jbc.M802826200 PMC2562052 18650420

[156] Kawasaki K Akashi S Shimazu R Yoshida T Miyake K Nishijima M. Mouse toll-like receptor 4.MD-2 complex mediates lipopolysaccharide-mimetic signal transduction by Taxol. J Biol Chem. (2000) 275:2251–4. doi: 10.1074/jbc.275.4.2251 10644670

[157] Kawasaki K Akashi S Shimazu R Yoshida T Miyake K Nishijima M. Involvement of TLR4/MD-2 complex in species-specific lipopolysaccharide-mimetic signal transduction by Taxol. J Endotoxin Res. (2001) 7:232–6. doi: 10.1177/09680519010070030701 11581576

[158] Kawasaki K Gomi K Nishijima M. Cutting edge: Gln22 of mouse MD-2 is essential for species-specific lipopolysaccharide mimetic action of taxol. J Immunol. (2001) 166:11–4. doi: 10.4049/jimmunol.166.1.11 11123270

[159] Kawasaki K Gomi K Kawai Y Shiozaki M Nishijima M. Molecular basis for lipopolysaccharide mimetic action of Taxol and flavolipin. J Endotoxin Res. (2003) 9:301–7. doi: 10.1177/09680519030090050501 14577846

[160] Millrud CR Mehmeti M Leandersson K. Docetaxel promotes the generation of anti-tumorigenic human macrophages. Exp Cell Res. (2018) 362:525–31. doi: 10.1016/j.yexcr.2017.12.018 29269075

[161] Tsavaris N Kosmas C Vadiaka M Kanelopoulos P Boulamatsis D. Immune changes in patients with advanced breast cancer undergoing chemotherapy with taxanes. Br J Cancer. (2002) 87:21–7. doi: 10.1038/sj.bjc.6600347 PMC2364288 12085250

[162] Manthey CL Perera PY Salkowski CA Vogel SN. Taxol provides a second signal for murine macrophage tumoricidal activity. J Immunol. (1994) 152:825–31. doi: 10.4049/jimmunol.152.2.825 7506736

[163] Guan W-L He Y Xu R-H. Gastric cancer treatment: recent progress and future perspectives. J Hematol Oncol. (2023) 16:57. doi: 10.1186/s13045-023-01451-3 37245017 PMC10225110

[164] Kim R An M Lee H Mehta A Heo YJ Kim K-M. Early tumor-immune microenvironmental remodeling and response to first-line fluoropyrimidine and platinum chemotherapy in advanced gastric cancer. Cancer Discovery. (2022) 12:984–1001. doi: 10.1158/2159-8290.CD-21-0888 34933901 PMC9387589

[165] An M Mehta A Min BH Heo YJ Wright SJ Parikh M. Early immune remodeling steers clinical response to first-line chemoimmunotherapy in advanced gastric cancer. Cancer Discovery. (2024) 14:766–85. doi: 10.1158/2159-8290.CD-23-0857 PMC11061611 38319303

[166] Ahlmann M Hempel G. The effect of cyclophosphamide on the immune system: implications for clinical cancer therapy. Cancer Chemother Pharmacol. (2016) 78:661–71. doi: 10.1007/s00280-016-3152-1 27646791

[167] Doloff JC Waxman DJ. VEGF receptor inhibitors block the ability of metronomically dosed cyclophosphamide to activate innate immunity-induced tumor regression. Cancer Res. (2012) 72:1103–15. doi: 10.1158/0008-5472.CAN-11-3380 PMC3294103 22237627

[168] Leong WI Ames RY Haverkamp JM Torres L Kline J Bans A. Low-dose metronomic cyclophosphamide complements the actions of an intratumoral C-class CpG TLR9 agonist to potentiate innate immunity and drive potent T cell-mediated anti-tumor responses. Oncotarget. (2019) 10:7220–37. doi: 10.18632/oncotarget.v10i68 PMC6944447 31921384

[169] Wu J Waxman DJ. Metronomic cyclophosphamide eradicates large implanted GL261 gliomas by activating antitumor Cd8(+) T-cell responses and immune memory. Oncoimmunology. (2015) 4:e1005521. doi: 10.1080/2162402X.2015.1005521 26137402 PMC4485826

[170] Lossos C Liu Y Kolb KE Christie AL Van Scoyk A Prakadan SM. Mechanisms of lymphoma clearance induced by high-dose alkylating agents. Cancer Discovery. (2019) 9:944–61. doi: 10.1158/2159-8290.CD-18-1393 PMC6606344 31040105

[171] Pallasch CP Leskov I Braun CJ Vorholt D Drake A Soto-Feliciano YM. Sensitizing protective tumor microenvironments to antibody-mediated therapy. Cell. (2014) 156:590–602. doi: 10.1016/j.cell.2013.12.041 24485462 PMC3975171

[172] Roghanian A Hu G Fraser C Singh M Foxall RB Meyer MJ. Cyclophosphamide enhances cancer antibody immunotherapy in the resistant bone marrow niche by modulating macrophage fcγR expression. Cancer Immunol Res. (2019) 7:1876–90. doi: 10.1158/2326-6066.CIR-18-0835 PMC7780711 31451483

[173] Roodhart JM Langenberg MH Vermaat JS Lolkema MP Baars A Giles RH. Late release of circulating endothelial cells and endothelial progenitor cells after chemotherapy predicts response and survival in cancer patients. Neoplasia. (2010) 12:87–94. doi: 10.1593/neo.91460 20072657 PMC2805887

[174] Daenen LGM Houthuijzen JM Cirkel GA Roodhart JML Shaked Y Voest EE. Treatment-induced host-mediated mechanisms reducing the efficacy of antitumor therapies. Oncogene. (2014) 33:1341–7. doi: 10.1038/onc.2013.94 23524584

[175] Roodhart JML He H Daenen LGM Monvoisin A Barber CL van Amersfoort M. Notch1 regulates angio-supportive bone marrow-derived cells in mice: relevance to chemoresistance. Blood. (2013) 122:143–53. doi: 10.1182/blood-2012-11-459347 PMC3701902 23690447

[176] DeNardo DG Brennan DJ Rexhepaj E Ruffell B Shiao SL Madden SF. Leukocyte complexity predicts breast cancer survival and functionally regulates response to chemotherapy. Cancer Discovery. (2011) 1:54–67. doi: 10.1158/2159-8274.CD-10-0028 22039576 PMC3203524

[177] Hughes R Qian B-Z Rowan C Muthana M Keklikoglou I Olson OC. Perivascular M2 macrophages stimulate tumor relapse after chemotherapy. Cancer Res. (2015) 75:3479–91. doi: 10.1158/0008-5472.CAN-14-3587 PMC5024531 26269531

[178] De Palma M Venneri MA Galli R Sergi Sergi L Politi LS Sampaolesi M. Tie2 identifies a hematopoietic lineage of proangiogenic monocytes required for tumor vessel formation and a mesenchymal population of pericyte progenitors. Cancer Cell. (2005) 8:211–26. doi: 10.1016/j.ccr.2005.08.002 16169466

[179] Mazzieri R Pucci F Moi D Zonari E Ranghetti A Berti A. Targeting the ANG2/TIE2 axis inhibits tumor growth and metastasis by impairing angiogenesis and disabling rebounds of proangiogenic myeloid cells. Cancer Cell. (2011) 19:512–26. doi: 10.1016/j.ccr.2011.02.005 21481792

[180] Sanchez LR Borriello L Entenberg D Condeelis JS Oktay MH Karagiannis GS. The emerging roles of macrophages in cancer metastasis and response to chemotherapy. J Leukoc Biol. (2019) 106:259–74. doi: 10.1002/JLB.MR0218-056RR PMC6779158 30720887

[181] Valind A Verhoeven BM Enoksson J Karlsson J Christensson G Mañas A. Macrophage infiltration promotes regrowth in MYCN-amplified neuroblastoma after chemotherapy. Oncoimmunology. (2023) 12:2184130. doi: 10.1080/2162402X.2023.2184130 36875552 PMC9980604

[182] Zhang L Qi Y Min H Ni C Wang F Wang B. Cooperatively responsive peptide nanotherapeutic that regulates angiopoietin receptor tie2 activity in tumor microenvironment to prevent breast tumor relapse after chemotherapy. ACS Nano. (2019) 13:5091–102. doi: 10.1021/acsnano.8b08142 30986342

[183] Jayatilleke KM Hulett MD. Heparanase and the hallmarks of cancer. J Transl Med. (2020) 18:453. doi: 10.1186/s12967-020-02624-1 33256730 PMC7706218

[184] Mohan CD Hari S Preetham HD Rangappa S Barash U Ilan N. Targeting heparanase in cancer: inhibition by synthetic, chemically modified, and natural compounds. iScience. (2019) 15:360–90. doi: 10.1016/j.isci.2019.04.034 PMC6548846 31103854

[185] Shafat I Barak AB Postovsky S Elhasid R Ilan N Vlodavsky I. Heparanase levels are elevated in the plasma of pediatric cancer patients and correlate with response to anticancer treatment. Neoplasia. (2007) 9:909–16. doi: 10.1593/neo.07673 PMC2077882 18030359

[186] Alishekevitz D Gingis-Velitski S Kaidar-Person O Gutter-Kapon L Scherer SD Raviv Z. Macrophage-induced lymphangiogenesis and metastasis following paclitaxel chemotherapy is regulated by VEGFR3. Cell Rep. (2016) 17:1344–56. doi: 10.1016/j.celrep.2016.09.083 PMC5098117 27783948

[187] Cava E Marzullo P Farinelli D Gennari A Saggia C Riso S. Breast cancer diet “BCD”: A review of healthy dietary patterns to prevent breast cancer recurrence and reduce mortality. Nutrients. (2022) 14:1-15. doi: 10.3390/nu14030476 PMC8839871 35276833

[188] De Cicco P Catani MV Gasperi V Sibilano M Quaglietta M Savini I. Nutrition and breast cancer: A literature review on prevention, treatment and recurrence. Nutrients. (2019) 11:1-28. doi: 10.3390/nu11071514 PMC6682953 31277273

[189] Ang HL Mohan CD Shanmugam MK Leong HC Makvandi P Rangappa KS. Mechanism of epithelial-mesenchymal transition in cancer and its regulation by natural compounds. Med Res Rev. (2023) 43:1141–200. doi: 10.1002/med.21948 36929669

[190] Avila-Carrasco L Majano P Sánchez-Toméro JA Selgas R López-Cabrera M Aguilera A. Natural plants compounds as modulators of epithelial-to-mesenchymal transition. Front Pharmacol. (2019) 10:715. doi: 10.3389/fphar.2019.00715 31417401 PMC6682706

[191] Carlsen MH Halvorsen BL Holte K Bøhn SK Dragland S Sampson L. The total antioxidant content of more than 3100 foods, beverages, spices, herbs and supplements used worldwide. Nutr J. (2010) 9:3. doi: 10.1186/1475-2891-9-3 20096093 PMC2841576

[192] Latronico T Petraglia T Sileo C Bilancia D Rossano R Liuzzi GM. Inhibition of MMP-2 and MMP-9 by dietary antioxidants in THP-1 macrophages and sera from patients with breast cancer. Molecules. (2024) 29:1-15. doi: 10.3390/molecules29081718 PMC11051835 38675538

[193] Shah R Ibis B Kashyap M Boussiotis VA. The role of ROS in tumor infiltrating immune cells and cancer immunotherapy. Metabolism. (2024) 151:155747. doi: 10.1016/j.metabol.2023.155747 38042522 PMC10872310

[194] Aggarwal V Tuli HS Varol A Thakral F Yerer MB Sak K. Role of reactive oxygen species in cancer progression: molecular mechanisms and recent advancements. Biomolecules. (2019) 9:1-26. doi: 10.3390/biom9110735 PMC6920770 31766246

[195] Nelson KK Melendez JA. Mitochondrial redox control of matrix metalloproteinases. Free Radic Biol Med. (2004) 37:768–84. doi: 10.1016/j.freeradbiomed.2004.06.008 15304253

[196] Przybyłek I Karpiński TM. Antibacterial properties of propolis. Molecules. (2019) 24:1-17. doi: 10.3390/molecules24112047 PMC6600457 31146392

[197] Zulhendri F Chandrasekaran K Kowacz M Ravalia M Kripal K Fearnley J. Antiviral, antibacterial, antifungal, and antiparasitic properties of propolis: A review. Foods. (2021) 10:1-29. doi: 10.3390/foods10061360 PMC8231288 34208334

[198] Wagh VD. Propolis: a wonder bees product and its pharmacological potentials. Adv Pharmacol Sci. (2013) 2013:308249. doi: 10.1155/2013/308249 24382957 PMC3872021

[199] Frión-Herrera Y Gabbia D Scaffidi M Zagni L Cuesta-Rubio O De Martin S. Cuban brown propolis interferes in the crosstalk between colorectal cancer cells and M2 macrophages. Nutrients. (2020) 12:1-16. doi: 10.3390/nu12072040 PMC7400951 32660099

[200] Batiha GE-S Alkazmi LM Wasef LG Beshbishy AM Nadwa EH Rashwan EK. (Myrtaceae): traditional uses, bioactive chemical constituents, pharmacological and toxicological activities. Biomolecules. (2020) 10:1-16. doi: 10.3390/biom10020202 PMC7072209 32019140

[201] Woo J-H Ahn J-H Jang DS Lee K-T Choi J-H. Effect of kumatakenin isolated from cloves on the apoptosis of cancer cells and the alternative activation of tumor-associated macrophages. J Agric Food Chem. (2017) 65:7893–9. doi: 10.1021/acs.jafc.7b01543 28763204

[202] Aldinucci D Borghese C Casagrande N. The CCL5/CCR5 axis in cancer progression. Cancers (Basel). (2020) 12:1-30. doi: 10.3390/cancers12071765 PMC7407580 32630699

[203] Xu M Wang Y Xia R Wei Y Wei X. Role of the CCL2-CCR2 signalling axis in cancer: Mechanisms and therapeutic targeting. Cell Prolif. (2021) 54:e13115. doi: 10.1111/cpr.v54.10 34464477 PMC8488570

[204] Cesari M Incalzi RA Zamboni V Pahor M. Vitamin D hormone: a multitude of actions potentially influencing the physical function decline in older persons. Geriatr Gerontol Int. (2011) 11:133–42. doi: 10.1111/j.1447-0594.2010.00668.x PMC4384440 21134097

[205] Lopes N Paredes J Costa JL Ylstra B Schmitt F. Vitamin D and the mammary gland: a review on its role in normal development and breast cancer. Breast Cancer Res. (2012) 14:211. doi: 10.1186/bcr3178 22676419 PMC3446331

[206] Vanhevel J Verlinden L Doms S Wildiers H Verstuyf A. The role of vitamin D in breast cancer risk and progression. Endocr Relat Cancer. (2022) 29:R33–r55. doi: 10.1530/ERC-21-0182 34935629

[207] Guo Y Jiang F Yang W Shi W Wan J Li J. Effect of 1α,25(OH)(2)D(3)-treated M1 and M2 macrophages on cell proliferation and migration ability in ovarian cancer. Nutr Cancer. (2022) 74:2632–43. doi: 10.1080/01635581.2021.2014903 34894920

[208] Liang P Henning SM Guan J Grogan T Elashoff D Cohen P. Effect of dietary omega-3 fatty acids on castrate-resistant prostate cancer and tumor-associated macrophages. Prostate Cancer Prostatic Dis. (2020) 23:127–35. doi: 10.1038/s41391-019-0168-8 PMC7031053 31439889

[209] Abdelaal M le Roux CW Docherty NG. Morbidity and mortality associated with obesity. Ann Transl Med. (2017) 5:161. doi: 10.21037/atm.2017.03.107 28480197 PMC5401682

[210] Pati S Irfan W Jameel A Ahmed S Shahid RK. Obesity and cancer: A current overview of epidemiology, pathogenesis, outcomes, and management. Cancers (Basel). (2023) 15:1-21. doi: 10.3390/cancers15020485 PMC9857053 36672434

[211] Jiralerspong S Goodwin PJ. Obesity and breast cancer prognosis: evidence, challenges, and opportunities. J Clin Oncol. (2016) 34:4203–16. doi: 10.1200/JCO.2016.68.4480 27903149

[212] Ewertz M Jensen MB Gunnarsdóttir K Højris I Jakobsen EH Nielsen D. Effect of obesity on prognosis after early-stage breast cancer. J Clin Oncol. (2011) 29:25–31. doi: 10.1200/JCO.2010.29.7614 21115856

[213] Ioannides SJ Barlow PL Elwood JM Porter D. Effect of obesity on aromatase inhibitor efficacy in postmenopausal, hormone receptor-positive breast cancer: a systematic review. Breast Cancer Res Treat. (2014) 147:237–48. doi: 10.1007/s10549-014-3091-7 25119728

[214] Karatas F Erdem GU Sahin S Aytekin A Yuce D Sever AR. Obesity is an independent prognostic factor of decreased pathological complete response to neoadjuvant chemotherapy in breast cancer patients. Breast. (2017) 32:237–44. doi: 10.1016/j.breast.2016.05.013 27318645

[215] Sartipy P Loskutoff DJ. Monocyte chemoattractant protein 1 in obesity and insulin resistance. Proc Natl Acad Sci U S A. (2003) 100:7265–70. doi: 10.1073/pnas.1133870100 PMC165864 12756299

[216] Weisberg SP McCann D Desai M Rosenbaum M Leibel RL Ferrante AW Jr. Obesity is associated with macrophage accumulation in adipose tissue. J Clin Invest. (2003) 112:1796–808. doi: 10.1172/JCI200319246 PMC296995 14679176

[217] Maliniak ML Miller-Kleinhenz J Cronin-Fenton DP Lash TL Gogineni K Janssen EAM. Crown-like structures in breast adipose tissue: early evidence and current issues in breast cancer. Cancers (Basel). (2021) 13:1-27. doi: 10.3390/cancers13092222 PMC8124644 34066392

[218] Birts CN Savva C Laversin SA Lefas A Krishnan J Schapira A. Prognostic significance of crown-like structures to trastuzumab response in patients with primary invasive HER2 + breast carcinoma. Sci Rep. (2022) 12:7802. doi: 10.1038/s41598-022-11696-6 35610242 PMC9130517

[219] Lacasa D Keophiphath M Miranville A Clement K. Macrophage-secreted factors impair human adipogenesis: involvement of proinflammatory state in preadipocytes. Endocrinology. (2007) 148:868–77. doi: 10.1210/en.2006-0687 17082259

[220] Keophiphath M Achard V Henegar C Rouault C Clément K Lacasa D. Macrophage-secreted factors promote a profibrotic phenotype in human preadipocytes. Mol Endocrinol. (2009) 23:11–24. doi: 10.1210/me.2008-0183 18945811 PMC5419324

[221] Nazari SS Mukherjee P. An overview of mammographic density and its association with breast cancer. Breast Cancer. (2018) 25:259–67. doi: 10.1007/s12282-018-0857-5 PMC5906528 29651637

[222] Sun X Glynn DJ Hodson LJ Huo C Britt K Thompson EW. CCL2-driven inflammation increases mammary gland stromal density and cancer susceptibility in a transgenic mouse model. Breast Cancer Res. (2017) 19:4. doi: 10.1186/s13058-016-0796-z 28077158 PMC5225654

[223] Kim G Karadal-Ferrena B Qin J Sharma VP Oktay IS Lin Y. Racial disparity in tumor microenvironment and distant recurrence in residual breast cancer after neoadjuvant chemotherapy. NPJ Breast Cancer. (2023) 9:52. doi: 10.1038/s41523-023-00547-w 37311792 PMC10264351

[224] Kim G Pastoriza JM Condeelis JS Sparano JA Filippou PS Karagiannis GS. The contribution of race to breast tumor microenvironment composition and disease progression. Front Oncol. (2020) 10:1022. doi: 10.3389/fonc.2020.01022 32714862 PMC7344193

[225] Pickens A. Racial disparities in esophageal cancer. Thorac Surg Clin. (2022) 32:57–65. doi: 10.1016/j.thorsurg.2021.09.004 34801196

[226] Jani C Mouchati C Abdallah N Mariano M Jani R Salciccioli JD. Trends in prostate cancer mortality in the United States of America, by state and race, from 1999 to 2019: estimates from the centers for disease control WONDER database. Prostate Cancer Prostatic Dis. (2023) 26:552–62. doi: 10.1038/s41391-022-00628-0 36522462

[227] Chornokur G Dalton K Borysova ME Kumar NB. Disparities at presentation, diagnosis, treatment, and survival in African American men, affected by prostate cancer. Prostate. (2011) 71:985–97. doi: 10.1002/pros.21314 PMC3083484 21541975

[228] Sparano JA Wang M Zhao F Stearns V Martino S Ligibel JA. Race and hormone receptor-positive breast cancer outcomes in a randomized chemotherapy trial. J Natl Cancer Inst. (2012) 104:406–14. doi: 10.1093/jnci/djr543 PMC3295746 22250182

[229] Nettey OS Walker AJ Keeter MK Singal A Nugooru A Martin IK. Self-reported Black race predicts significant prostate cancer independent of clinical setting and clinical and socioeconomic risk factors. Urol Oncol. (2018) 36:501.e1–.e8. doi: 10.1016/j.urolonc.2018.06.011 PMC6214716 30236853

[230] Pastoriza JM Karagiannis GS Lin J Lanjewar S Entenberg D Condeelis JS. Black race and distant recurrence after neoadjuvant or adjuvant chemotherapy in breast cancer. Clin Exp Metastasis. (2018) 35:613–23. doi: 10.1007/s10585-018-9932-8 PMC6202136 30136072

[231] Martin DN Boersma BJ Yi M Reimers M Howe TM Yfantis HG. Differences in the tumor microenvironment between African-American and European-American breast cancer patients. PloS One. (2009) 4:e4531. doi: 10.1371/journal.pone.0004531 19225562 PMC2638012

[232] Koru-Sengul T Santander AM Miao F Sanchez LG Jorda M Glück S. Breast cancers from black women exhibit higher numbers of immunosuppressive macrophages with proliferative activity and of crown-like structures associated with lower survival compared to non-black Latinas and Caucasians. Breast Cancer Res Treat. (2016) 158:113–26. doi: 10.1007/s10549-016-3847-3 PMC5129629 27283835

